# Highly DUV to NIR-II responsive broadband quantum dots heterojunction photodetectors by integrating quantum cutting luminescent concentrators

**DOI:** 10.1038/s41377-024-01604-0

**Published:** 2024-10-15

**Authors:** Nan Ding, Wen Xu, Hailong Liu, Yuhan Jing, Zewen Wang, Yanan Ji, Jinlei Wu, Long Shao, Ge Zhu, Bin Dong

**Affiliations:** Key Laboratory of New Energy and Rare Earth Resource Utilization of State Ethnic Affairs Commission, School of Physics and Materials Engineering, Dalian Minzu University, 18 Liaohe West Road, Dalian, 116600 China

**Keywords:** Photonic devices, Imaging and sensing

## Abstract

Low-cost, high-performance, and uncooled broadband photodetectors (PDs) have potential applications in optical communication etc., but it still remains a huge challenge to realize deep UV (DUV) to the second near-infrared (NIR-II) detection for a single broadband PD. Herein, a single PD affording broadband spectral response from 200 to 1700 nm is achieved with a vertical configuration based on quantum dots (QDs) heterojunction and quantum cutting luminescent concentrators (QC–LC). A broadband quantum dots heterojunction as absorption layer was designed by integrating CsPbI_3_:Ho^3+^ perovskite quantum dots (PQDs) and PbS QDs to realize the spectral response from 400 to 1700 nm. The QC–LC by employing CsPbCl_3_:Cr^3+^, Ce^3+^, Yb^3+^, Er^3+^ PQDs as luminescent conversion layer to collect and concentrate photon energy for boosting the DUV–UV (200–400 nm) photons response of PDs by waveguide effect. Such broadband PD displays good stability, and outstanding sensitivity with the detectivity of 3.19 × 10^12^ Jones at 260 nm, 1.05 × 10^13^ Jones at 460 nm and 2.23 × 10^12^ Jones at 1550 nm, respectively. The findings provide a new strategy to construct broadband detector, offering more opportunities in future optoelectronic devices.

## Abstract (web summary)

Highly DUV to NIR-II responsive broadband quantum dots heterojunction photodetectors.

## Graphical abstract

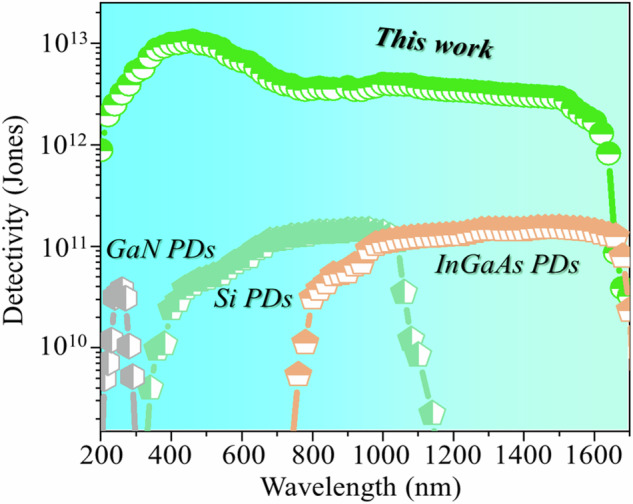

## Introduction

Photodetectors (PDs) with the ability to capture optical signals and convert them into electrical signals, are significance in optoelectronic applications^1–4^. Particularly, uncooled broadband PDs capable of covering the near infrared (NIR) region with low-cost and high-performance are vital components for optical communication, national security, military monitoring, and bioimaging^5–7^. Currently, the commercial broadband PDs were fabricated by mature materials with narrow bandgap, such as silicon (Si), indium gallium arsenide (InGaAs), and mercury cadmium telluride (HgCdTe) etc. With the rapid development of detection technology, they achieved excellent response within visible to short-wave-NIR (eg., Si PDs)^8^ or the second near-infrared (NIR-II) region (eg., InGaAs PDs)^9^. However, to our knowledge, few of them are targeted to realize high performance in the whole region spanning from deep ultraviolet (DUV) to NIR-II. Previously, to construct fully spectral responsive PDs, two or more PDs should be integrated into one system, inevitably bringing about large size, high price, and especially for incomparable responsiveness among different PDs. Meanwhile, these PDs usually encounter with complex fabrication process and low temperature working conditions etc. All of these cause huge difficulties in practical applications.

All inorganic lead halide (CsPbX_3_, X = Cl, Br or I) perovskite quantum dots (PQDs) have attracted numerous attention in solar cells, light-emitting diodes (LEDs), lasers and PDs, owing to their excellent light sensitivity, tunable bandgap, large absorption coefficient, and high collection efficiency of carriers^10–16^. Especially, iodine based PQDs process narrow bandgap (∼1.73 eV) with a wide spectral response, exhibiting a great potential for photoactive layer to constructing broadband PDs^17–19^. Nevertheless, the current PDs based on CsPbI_3_ PQDs still suffer from low sensitivity, slow response time, and poor stability, due to the high trap density and susceptibility to UV light and oxygen^20–22^. In addition, its responsive wavelength was concentrated in UV–Visible region (350 nm–750 nm), uncapable of covering the DUV–UV (200 nm–350 nm) and NIR (>750 nm) because of the insensitivity for DUV–UV light and limitation of bandgap^23,24^. Therefore, how to overcome the above issues as the main challenges for obtaining broadband and high performance PDs.

Doping with lanthanide ions (eg.,Ce^3+^, Eu^3+^, Eu^2+^, Sm^3+^, Nd^3+^, Er^3+^) has emerged to boost the optical and electrical performance of PQDs, such as improving the photoluminescence quantum yields (PLQYs), enhancing UV- and long term- stability, and decreasing the trap density^25–29^. It is mainly assigned to their unique spectroscopic properties and atomic radii suitable for the tolerance factor of PQDs^13,27,30–32^. Meanwhile, a hybrid structure combining perovskites with the organic or inorganic narrower bandgap semiconductor materials (e.g.,BTP-4Cl:PBDB-TF, PbSe, HgTe, PbS) is one of a promising strategy to achieve high performance broadband PDs with NIR spectral response^33–35^. Among them, lead sulfide (PbS) quantum dots (QDs) with easily adjustable absorption range to the NIR-II region via the size dependent quantum confinement^36,37^. Besides, it has large absorption coefficient (∼10^6 ^ M^-1^ cm^-1^) and better chemical stability^38^.

Aiming at the low DUV–UV response of PDs, the downshifting luminescence materials provide an effective route to convert DUV–UV light into visible or NIR photons, and subsequently being recaptured by the integrated PDs^8,23^. Various luminescent conversion materials including the quantum cutting materials with the PLQYs of ~200% have been implemented to significantly improve the UV response of solar cells or PDs (eg., Si and perovskites)^39,40^. But the huge photon energy loss by directly integrating with photoelectric devices always happens, stemming from the random emission direction of luminescent conversion materials. Luminescent concentrator (LC) devices with directional emission consisting of transparent polymer sheets doped with luminescent species can collect and concentrate photon energy to minimize the photons loss through waveguide effect.

In this work, high PLQYs (93.5%) and stability, and less defects of CsPbI_3_:Ho^3+^ PQDs were synthesized, served as the UV–Visible photoresponsivity layer for broadband PDs. To broaden the NIR-II response of broadband PDs, the PbS QDs with Visible-NIR absorption region were directly assembled on the surface of CsPbI_3_:Ho^3+^ PQDs to form a heterojunction composite. Meanwhile, the Cr^3+^, Ce^3+^, Yb^3+^, Er^3+^ doped CsPbCl_3_ PQDs with the high PLQYs (~179%) were incorporated into the polymethyl methacrylate to prepare a quantum cutting LC (QC–LC), which can convert DUV–UV light into NIR photons and subsequently being recaptured by the integrated PDs. This broadband PDs consists of QC–LC and CsPbI_3_:Ho^3+^ - PbS QDs heterojunction to realize the full spectrum response spanning from 200 nm–1700 nm, showing the detectivity of 3.19 × 10^12^ Jones at 260 nm, 1.05 × 10^13^ Jones at 460 nm, and 2.23 × 10^12^ Jones at 1550 nm. Furthermore, this broadband PDs demonstrate outstanding air- and UV- stability and high-contrast imaging applications.

## Results

**Fig. 1:**
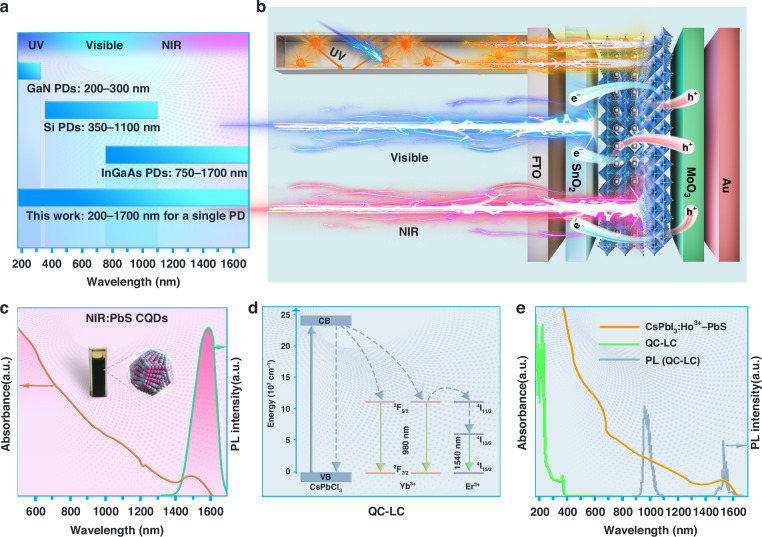
DUV—NIR-II broadband PD. **a** The detection range for the commercial GaN PDs, Si PDs, InGaAs PDs, and this work. **b** Schematic of a broadband PD configuration. **c** Absorption and emission spectra of PbS QDs. Inset is the image of PbS QDs. **d** Energy level diagram of QC–LC. **e** Absorption spectra of QC–LC and CsPbI_3_:Ho^3+^—PbS QDs, and emission spectra of QC–LC

In general, the commercial broadband PDs (UV–NIR) usually by integrating two or more PDs (eg., GaN, Si, and InGaAs) for detecting DUV to UV, Visible to short-wave—NIR, and NIR light (Fig. 1a), respectively, which can bring complex fabrication process and higher costs. Herein, we successfully fabricated a broadband responsive PD from DUV to NIR-II (200 nm–1700 nm) with the device structures of CsPbCl_3_:Cr^3+^, Ce^3+^, Yb^3+^, Er^3+^ PQDs doped polymethyl methacrylate QC–LC / FTO / SnO_2_ / CsPbI_3_:Ho^3+^ - PbS QDs heterojunction / MoO_3_ / Au, as shown in Fig. 1b. The corresponding cross-sectional scanning electron microscope (SEM) image of the broadband PD is displayed in Fig. S1. In such devices, the CsPbI_3_:Ho^3+^ PQDs with outstanding optoelectronic performance is responsible for UV–Visible light (350 nm–700 nm). The PbS QDs were prepared through hot injection method exhibits broadband absorption from 500 nm to 1700 nm (Fig. 1c). Then, they were assembled on the surface of CsPbI_3_:Ho^3+^ PQDs via mechanical stirring, serving as the NIR-II absorption material. Finally, QC–LC with the strong absorption from 200 nm to 400 nm and efficiently converts them into typical NIR emission of Yb^3+^ and Er^3+^ through quantum cutting process (Fig. 1d). Consequently, these NIR photons can be further reabsorbed by the CsPbI_3_:Ho^3+^—PbS QDs heterojunction, thereby expanding the response in DUV to UV region (Fig. 1e).

**Fig. 2:**
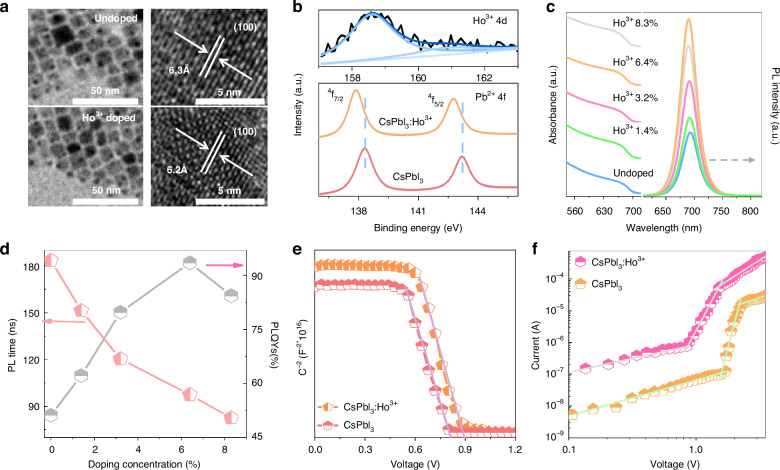
Ho3+ doped CsPbI3 PQDs. **a** TEM and HR-TEM (right) of undoped CsPbI_3_ and CsPbI_3_:Ho^3+^ (6.4%) PQDs. **b** XPS spectra of Ho^3+^ (4*d*) and Pb^2+^ (4 *f*) of undoped CsPbI_3_ and CsPbI_3_:Ho^3+^ (6.4%) PQDs. Absorption and emission spectra (**c**), and PLQYs and PL lifetime (**d**) of undoped CsPbI_3_ and CsPbI_3_:Ho^3+^ PQDs with different Ho^3+^ concentrations. **e** Mott–Schottky of CsPbI_3_ and CsPbI_3_:Ho^3+^ (6.4%) PQDs. **f** I-V curves with electron-only devices based on CsPbI_3_ and CsPbI_3_:Ho^3+^ (6.4%) PQDs

To obtain the high performance and broadband response in a single PD, a series of optical and electrical experiments were conducted. Firstly, to improve the performance of PQDs, lanthanide ions (Ho^3+^) was selected as dopant to incorporate into CsPbI_3_ PQDs prepared by modified hot injection method^41–43^. As revealed in the transmission electron microscope (TEM) and high-resolution TEM (HR-TEM) images (Fig. 2a and S2–S4), the undoped CsPbI_3_ and CsPbI_3_:Ho^3+^ PQDs with the similar cubic shape were obtained. The average size of CsPbI_3_ PQDs is ~11.6 nm, which gradually decreases to ~11.4 nm, ~11.1 nm, ~10.8 nm and ~10.5 nm with increasing Ho^3+^ concentrations from 1.4% to 8.3%, respectively (Fig. S5). The practical Ho^3+^ doping concentration was identified by the inductively coupled plasma optical emission spectrometry (ICP–OES) and the X-ray photoelectron spectroscopy (XPS) (Table S1). The lattice distances of the (100) plane of undoped CsPbI_3_ and CsPbI_3_:Ho^3+^ PQDs are determined to be ~6.3 Å and ~6.2 Å. The lattice shrinkage is ascribed to the replacement of the larger radius of Pb^2+^ (~119 pm) by smaller size of Ho^3+^ (~90.1 pm)^44–46^. The energy dispersive X-ray (EDX) mapping images demonstrate that all of the elements (cesium, lead, iodine, and holmium) exist in CsPbI_3_:Ho^3+^ PQDs (Fig. S6). The X-ray diffraction (XRD) patterns in Fig. S7 shows that undoped CsPbI_3_ and CsPbI_3_:Ho^3+^ PQDs have the same cubic structure without impurity peak, and the (100) peaks of PQDs gradually shift to higher diffraction angle after Ho^3+^ doping. Meanwhile, the XPS survey spectra evidence that the appearance of Ho^3+^ 4*d* peaks in CsPbI_3_:Ho^3+^ PQDs, and the peaks of Cs^+^ 3*d*, Pb^2+^ 4 *f*, and I^-^ 3*d* are presented in undoped CsPbI_3_ and CsPbI_3_:Ho^3+^ PQDs (Fig. 2b and S8). Compared with the undoped CsPbI_3_ PQDs, the binding energy of Pb^2+ 4^*f*_5/2_ and ^4^*f*_7/2_ moves to lower energy after Ho^3+^ doping, while Cs^+^ 3*d* and I^-^ 3*d* shows slight changes. Based on the above observations, we confirm that Ho^3+^ ions are successfully doped into the CsPbI_3_ PQDs and mainly replace the Pb^2+^ sites.

Figure 2c shows the absorption and emission spectra of pristine CsPbI_3_ and CsPbI_3_:Ho^3+^ PQDs with various Ho^3+^ doping concentration. The absorption peak of CsPbI_3_ PQDs gradually blue shifts from 683 nm to 678 nm with increasing Ho^3+^ concentration. In line with the absorption peak variation, the exciton bandgap increases from 1.76 eV of pristine CsPbI_3_ to 1.82 eV of CsPbI_3_:Ho^3+^ (6.4%) (Fig. S9). It can be mainly due to the lattice contraction by substituting Pb^2+^ with Ho^3+^ (Supplementary Note 1)^47,48^. Accordingly, the exciton emission peak of CsPbI_3_ PQDs moves to high energy from 693 nm to 685 nm with Ho^3+^ doping (Fig. S10). Significantly, after Ho^3+^ doping, the emission intensity of CsPbI_3_ PQDs rapidly enhances with the optimal Ho^3+^ concentration of 6.4%. The PLQYs of CsPbI_3_:Ho^3+^ PQDs reaches remarkable 93.5% (Fig. 2d). In addition, the exciton decay lifetimes gradually decrease from 183.5 ns of pristine CsPbI_3_ PQDs to 82.6 ns of CsPbI_3_:Ho^3+^ (8.3%) PQDs (Fig. S12). Then, the radiative rates (k_r_) and nonradiative rates (k_nr_) of CsPbI_3_ and CsPbI_3_:Ho^3+^ PQDs were calculated according to their PLQYs and PL decay lifetimes. As displayed in Table S2, the k_r_ of PQDs increases about 3.44 folds from 2.79 × 10^6^ S^-1^ of undoped CsPbI_3_ PQDs to 9.59 × 10^6^ S^-1^ of CsPbI_3_:Ho^3+^ (6.4%) PQDs. The k_nr_ of CsPbI_3_ PQDs is 2.66 × 10^6^ S^-1^, which decreases to 0.67 × 10^6^ S^-1^ of CsPbI_3_:Ho^3+^ (6.4%) PQDs. Compared to the undoped CsPbI_3_ PQDs, the k_r_ process becomes dominant for CsPbI_3_:Ho^3+^ PQDs, leading to the high emission efficiency of CsPbI_3_:Ho^3+^ (6.4%) PQDs. Based to the formula^44^:

1 $${k}_{r}=\frac{n{e}^{2}{{\rm{\omega }}}^{2}}{3{{\rm{\varepsilon }}}_{0}{m}_{0}{c}^{3}}{f}_{0}{\left(\frac{{\rm{\mu }}}{M}{E}_{b}\right)}^{1.5}\frac{1-{e}^{\frac{-\Delta E}{{kT}}}}{\Delta E}$$

where *μ*, *k*, *m*_*0*_, *ε*_*0*_, *ΔE*, *ω*, and n are the equivalent mass of exciton, the Boltzmann’s constant, the mass of exciton, the vacuum permittivity, the energy line width of PQDs, the optical transition frequency and the refractive index. The k_r_ can be mainly related to the exciton binding energy (*E*_*b*_) of PQDs. The *E*_*b*_ is achieved from the temperature-dependent PL intensity of PQDs by^21^:

2 $${I}_{(T)}=\frac{{I}_{0}}{1+A\exp \left(\frac{{-E}_{b}}{{kT}}\right)}$$

where the *I*_*0*_ is the PL intensity of PQDs at 0 k, A is the proportional constant. The E_b_ of CsPbI_3_:Ho^3+^ (6.4%) PQDs is 32.8 meV (Fig. S13), and 20 meV for pristine CsPbI_3_ PQDs^49^. On the basis of Eq. (1), it suggests that the increasing k_r_ with Ho^3+^ doping is mainly originates from the increase of the *E*_*b*_ of PQDs.

The role of Ho^3+^ on the electrical performance of PQDs films were investigated using I-V curves. As shown in Fig. S14 and Supplementary Note 2, the conductivity (σ) was determined to be 1.18 × 10^-6^ S cm^-1^ for the pristine CsPbI_3_ PQDs, which increases to 3.54 × 10^-6^ S cm^-1^ in CsPbI_3_:Ho^3+^ PQDs. The Mott–Schottky analysis was used to obtain the built-in potential (*V*_bi_) of the devices through capacitance-voltage (*C–V*) measurements (Fig. 2e and Supplementary Note 3). The CsPbI_3_:Ho^3+^ PQDs exhibits higher *V*_bi_ (~0.89 V) than that of the undoped CsPbI_3_ PQDs (~0.81 V), indicating the decreased carrier accumulation and improved carrier transports in PDs^50^. Meanwhile, according to the space charge limited current (SCLC) method, the trap density of undoped CsPbI_3_ and CsPbI_3_:Ho^3+^ PQDs were deduced to be 1.05 × 10^17 ^cm^-3^ and 4.08 × 10^16 ^ cm^-3^ (Fig. 2f and Supplementary Note 4). It reduces 2.57 folds of CsPbI_3_ PQDs with Ho^3+^ doping, dominating the decrease of k_nr_ for CsPbI_3_:Ho^3+^ PQDs. The charge carrier mobility increases from 6.35 × 10^-4^ cm^2^ V^-1^ S^-1^ of pristine CsPbI_3_ PQDs to 4.94 × 10^-3^ cm^2^ V^-1^ S^-1^ of CsPbI_3_:Ho^3+^ PQDs. Furthermore, the air- and UV- stability of CsPbI_3_ PQDs significantly improves after Ho^3+^ doping (Fig. S15). It can be seen that the emission intensity of undoped CsPbI_3_ PQDs degrades rapidly, almost disappears after 8 days storage and 12 h UV irradiation. In contrast, the emission intensity of CsPbI_3_:Ho^3+^ PQDs maintains about 95% and 90% of the original intensity after 50 days storage and 24 h UV radiation. The tolerance factor of CsPbI_3_:Ho^3+^ PQDs was estimated to be 0.813, larger than that 0.807 of pristine CsPbI_3_ PQDs (Fig. S16). Therefore, reduced trap density, boosted the carrier mobility and conductivity, enhanced the stability, and increased tolerance factor of CsPbI_3_ PQDs with Ho^3+^ doping account for the improved performance for broadband PDs based on CsPbI_3_:Ho^3+^ PQDs.

**Fig. 3:**
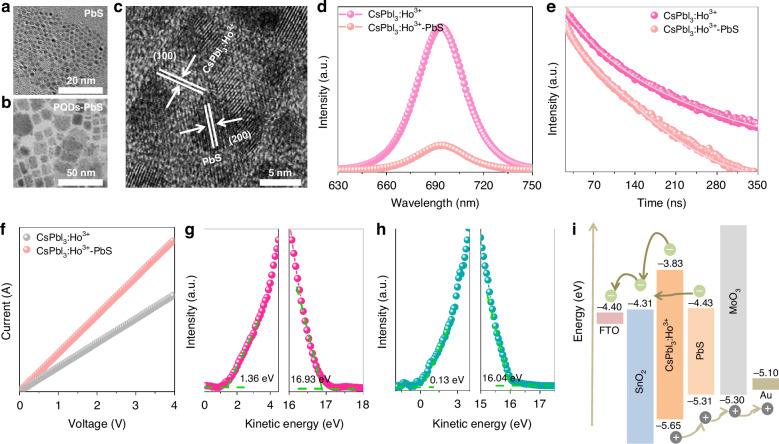
Photoelectric characteristics of CsPbI3:Ho3+- PbS QDs heterojunction. TEM images of PbS QDs (**a**) and CsPbI_3_:Ho^3+^—PbS QDs (**b**). **c** HR-TEM image of CsPbI_3_:Ho^3+^—PbS QDs. **d** Emission spectra of CsPbI_3_:Ho^3+^ PQDs and CsPbI_3_:Ho^3+^ - PbS QDs. **e** PL lifetime of CsPbI_3_:Ho^3+^ PQDs and CsPbI_3_:Ho^3+^—PbS QDs at 692 nm. **f** *I-V* curves of CsPbI_3_:Ho^3+^ PQDs and CsPbI_3_:Ho^3+^—PbS QDs. UPS curves of CsPbI_3_:Ho^3+^ PQDs (**g**) and PbS QDs (**h**). The Fermi level of CsPbI_3_:Ho^3+^ PQDs and PbS QDs are -4.29 eV and -5.18 eV, respectively. **i** Schematic illustration of the each layer in PDs

We next performed the optical and electrical analysis of CsPbI_3_:Ho^3+^ PQDs by combining with PbS QDs. As shown in Fig. 3a and S17–S18, the PbS QDs exhibits the cubic shape with an average diameter of ~4.4 nm were obtained by typical hot injection method^51^. Then, the smaller sized PbS QDs would attach on the CsPbI_3_:Ho^3+^ PQDs surface to formation of a heterojunction composite by electrostatic interactions (Fig. 3b and S19). We further revealed the HR-TEM of CsPbI_3_:Ho^3+^—PbS QDs heterojunction, in which the lattice plane of CsPbI_3_:Ho^3+^ (100) and PbS (200) can be clearly identified (Fig. 3c). The CsPbI_3_:Ho^3+^—PbS QDs heterojunction exhibits a complementary absorption in the visible to NIR-II region (400 nm–1700 nm), where the enhanced absorption within 400 nm–681 nm is corresponding to exciton absorption of CsPbI_3_:Ho^3+^ PQDs (Fig. S20). The PL intensity of CsPbI_3_:Ho^3+^ PQDs dramatically reduces after integrating with PbS QDs (Fig. 3d), accompanied by the shortening of exciton lifetime of CsPbI_3_:Ho^3+^ PQDs from 97.5 ns to 62.3 ns for CsPbI_3_:Ho^3+^—PbS QDs heterojunction (Fig. 3e). Those results indicate that the CsPbI_3_:Ho^3+^—PbS QDs heterojunction can effective improve the carrier extraction and transfer. Meanwhile, the NIR emission of PbS QDs slightly increases for CsPbI_3_:Ho^3+^—PbS QDs heterojunction under 808 nm excitation (Fig. S21), enabling the high responsivity of PDs in NIR region.

To further investigate the charge recombination dynamic of CsPbI_3_:Ho^3+^ PQDs after combining with the PbS QDs, the *I-V* curves of CsPbI_3_:Ho^3+^ PQDs and CsPbI_3_:Ho^3+^—PbS QDs heterojunction were measured (Fig. 3f). It can be seen that CsPbI_3_:Ho^3+^ - PbS QDs heterojunction obtains higher σ (5.11×10^-6^ S cm^-1^), compared with the CsPbI_3_:Ho^3+^ PQDs device (3.54 × 10^-6^ S cm^-1^). The ultraviolet photoelectron spectroscopy (UPS) was employed to determine the energy levels of the CsPbI_3_:Ho^3+^ PQDs and PbS QDs (Fig. 3g, h). The valence band maximum (VBM) and the conduction band minimum (CBM) of CsPbI_3_:Ho^3+^ PQDs and PbS QDs are deduced to be -5.65 eV and -5.31 eV, and -3.83 eV and -4.43 eV, respectively. As illustrated in Fig. 3i, the band alignment between CsPbI_3_:Ho^3+^ PQDs and PbS QDs enables them to form a heterojunction. Under visible light illumination, the generated electrons in CsPbI_3_:Ho^3+^ PQDs tends to enter electron transport layer (ETL, SnO_2_). While the holes move to PbS QDs, subsequently transfer to hole transport layer (HTL, MoO_3_). Meanwhile, the PbS QDs captures the NIR light to produce electrons and holes, the electrons transfer to ETL by CsPbI_3_:Ho^3+^ PQDs, and the holes directly enter into HTL. Thus, the PDs combining CsPbI_3_:Ho^3+^ PQDs with PbS QDs brings about the broadband response from visible to NIR-II.

**Fig. 4:**
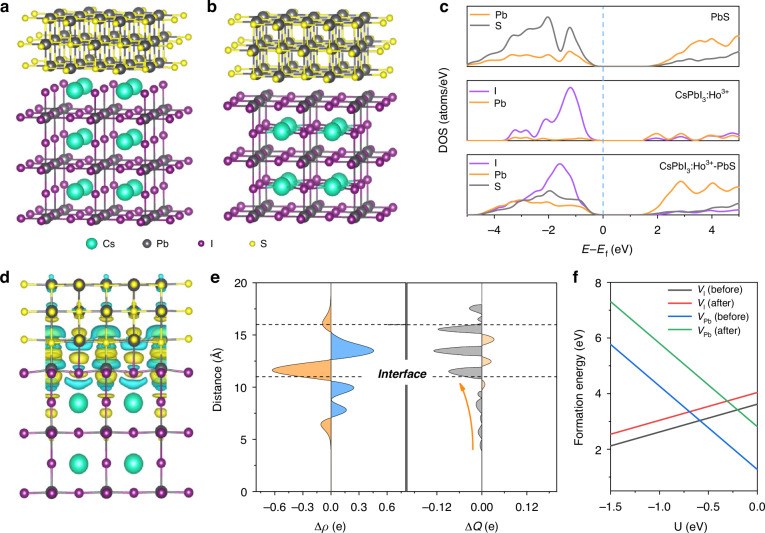
DFT calculations of CsPbI3:Ho3+- PbS QDs heterojunction. Optimized CsPbI_3_:Ho^3+^-PbS heterojunction with the (**a**) CsI / PbS and (**b**) PbI_2_ / PbS interfaces from side view. **c** Calculated DOS of the PbS, CsPbI_3_:Ho^3+^ and CsPbI_3_:Ho^3+^-PbS heterojunction. Charge density difference (**d**) *x*–*y* plane average charge difference and charge displacement curves along the *z*-direction of the PbI_2_ / PbS interface (**e**). **f** The Pb vacancies (*V*_*Pb*_) and I vacancies (*V*_*I*_) defect formation energies for the CsPbI_3_:Ho^3+^ and CsPbI_3_:Ho^3+^ - PbS heterojunction with respect to the chemical potential of the I atom

The structural and photophysical interaction between of CsPbI_3_:Ho^3+^ PQDs and PbS QDs were calculated through density functional theory (DFT) (Supplementary Note 5). Figure 4a, b shows the optimized CsPbI_3_:Ho^3+^—PbS QDs heterojunction with the PbI_2_ / PbS and CsI / PbS interfaces. The adhesive energies of PbI_2_ / PbS interface (-3.22 eV) is smaller than that of the CsI / PbS interface (-2.07 eV), indicating that the PbI_2_ / PbS is more stable interface structure^52^. The strong electron interface coupling between PbS and CsPbI_3_:Ho^3+^ is revealed by the density of states (DOS) functional (Fig. 4c), where the significant overlap between the Pb 6*p* and I 5*p* states in CsPbI_3_:Ho^3+^ PQDs with the S 2*p* orbital in PbS QDs. Furthermore, the bandgap of CsPbI_3_:Ho^3+^—PbS QDs heterojunction shows a slight smaller than that of the CsPbI_3_:Ho^3+^ PQDs, benefiting for the effectively transition of electrons from VBM to CBM. In order to understand the charge distribution of the CsPbI_3_:Ho^3+^—PbS QDs heterojunction interface, the charge density difference *Δρ* is obtained by^53^:

3 $$\varDelta {\rho }={{\rho }}_{{hete}}-{{\rho }}_{{PbS}}-{{\rho }}_{P{QDs}}$$

where *ρ*_*hete*_ is the charge density of the fully relaxed CsPbI_3_:Ho^3+^—PbS QDs heterojunction, *ρ*_*PbS*_and *ρ*_*PQDs*_ are the isolated PbS QDs and CsPbI_3_:Ho^3+^ PQDs slab. As shown in Fig. 4d, the charge depletions underneath Pb / I atoms for PbI_2_ / PbS interface, which is favorable for efficient charge migration from CsPbI_3_:Ho^3+^ PQDs to PbS QDs. Meanwhile, the *x*–*y* plane average charge difference *Δq* is calculated by^53^:

4 $$\varDelta q={\int }_{\!-{{\infty }}}^{{{\infty }}}{dy}{\int }_{\!-{{\infty }}}^{{{\infty }}}\Delta {\rm{\rho }}\,d{\chi }$$

In general, the charge accumulation and depletion can be obtained based on the positive and negative *Δq*, respectively^54^. Fig. 4e displays the *Δq* of PbI_2_ / PbS interface, where a dipole can be produced between the CsPbI_3_:Ho^3+^ PQDs surface and PbS QDs because of the charge accumulation and depletion, facilitating the charge separation and transport. Moreover, the charge displacement (CD) functional (*ΔQ*) can be achieved through integrating the *x*–*y* plane average charge difference Δq along the z direction^52^:

5 $$\varDelta Q={\int }_{\!-{{\infty }}}^{z}\varDelta q\,{dz}$$

where the positive *ΔQ* values indicates the charge transfer from the right to the left across the perpendicular plane through this direction and vice versa. The CD plot of PbI_2_ / PbS interface along z direction present that the whole negative for CsPbI_3_:Ho^3+^ PQDs side, which means that the charge transport from CsPbI_3_:Ho^3+^ PQDs to PbS QDs. As shown in Fig. 4f, we calculated the defect formation energies of Pb^2+^ vacancies (*V*_*Pb*_) and I^-^ vacancies (*V*_*I*_) in CsPbI_3_:Ho^3+^ PQDs and CsPbI_3_:Ho^3+^—PbS QDs heterojunction, where the *V*_*Pb*_ and *V*_*I*_ exhibit higher defect formation energies after PbS QDs coupling, benefiting for the responsivity of PDs^23^.

**Fig. 5:**
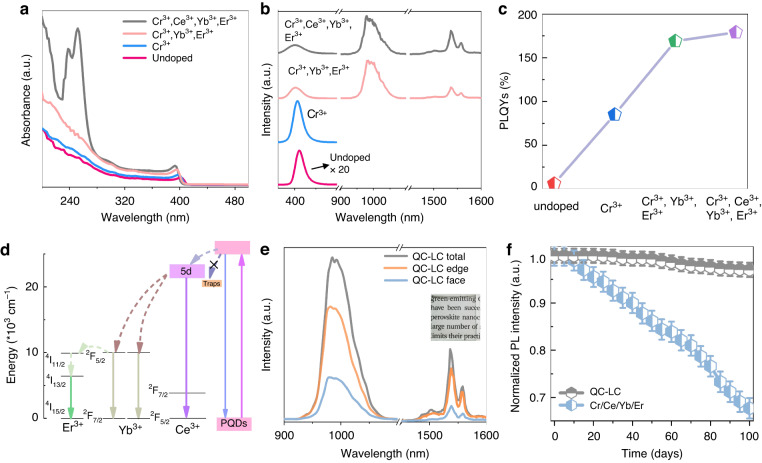
Optical characteristics of QC-LC. Absorption spectra (**a**), emission spectra (**b**), and PLQYs (**c**) of undoped, Cr^3+^, Cr^3+^ / Yb^3+^ / Er^3+^, and Cr^3+^ / Ce^3+^ / Yb^3+^ / Er^3+^ doped CsPbCl_3_ PQDs. **d** Energy level diagram of Cr^3+^ / Ce^3+^ / Yb^3+^ / Er^3+^ doped CsPbCl_3_ PQDs. **e** The emission spectra of QC–LC using an integrating sphere method. To accuracy obtained the face emission spectra of QC–LC, the black tapes with high light absorption was used to the edges of QC–LC. The inset is the picture of QC-LC. **f** Normalized PL intensity of QC–LC and Cr^3+^ / Ce^3+^ / Yb^3+^ / Er^3+^ doped CsPbCl_3_ PQDs films as a function of storage time

To fabricate QC–LC, the cubic and uniform Cr^3+^ / Ce^3+^ / Yb^3+^ / Er^3+^ doped CsPbCl_3_ PQDs with the average diameter of ~8.4 nm were prepared through the hot-injection method (Fig. S22)^55^. The Cr^3+^ and Ce^3+^ were selected to improve emission intensity and boost DUV absorption of CsPbCl_3_ PQDs. The emission of Yb^3+^ and Er^3+^ ions locate within the absorption of CsPbI_3_:Ho^3+^—PbS QDs heterojunction, and has high efficient quantum cutting behavior in CsPbCl_3_ PQDs. All the elements (Cs, Pb, Cl, Cr, Ce, Yb, and Er) exist in Cr^3+^ / Ce^3+^ / Yb^3+^ / Er^3+^ doped CsPbCl_3_ PQDs (Fig. S23). Figure 5a displays the UV–Visible absorption spectra of undoped, Cr^3+^ doped CsPbCl_3_ PQDs, Cr^3+^ / Yb^3+^ / Er^3+^ doped CsPbCl_3_ PQDs, and Cr^3+^ / Ce^3+^ / Yb^3+^ / Er^3+^ doped CsPbCl_3_ PQDs. It can be seen that the absorption peak of CsPbCl_3_ PQDs locates at 405 nm, and blue shifts to 391 nm after Cr^3+^ / Ce^3+^ / Yb^3+^ / Er^3+^ doping, ascribed to the lattice contraction of PQDs by substituting Pb^2+^ with the smaller size doping ions^47,56^. Moreover, the absorption spectra of Cr^3+^ / Ce^3+^ / Yb^3+^ / Er^3+^ doped CsPbCl_3_ PQDs present a significant enhancement within 200 nm–280 nm after Ce^3+^ doping, attributed to absorption contribution of 4 *f* - 5*d* for Ce^3+^^42^. Fig. 5b shows the emission spectra of undoped CsPbCl_3_ PQDs, CsPbCl_3_:Cr^3+^ PQDs, CsPbCl_3_:Cr^3+^, Yb^3+^, Er^3+^ PQDs, and CsPbCl_3_:Cr^3+^, Ce^3+^, Yb^3+^, Er^3+^ PQDs. It is found that an obvious exciton emission band centering at 410 nm for CsPbCl_3_ PQDs was observed under 365 nm excitation, which significant improvement after Cr^3+^ doping. Except for exciton emission, two additional emission peaks in CsPbCl_3_:Cr^3+^, Yb^3+^, Er^3+^ and CsPbCl_3_:Cr^3+^, Ce^3+^, Yb^3+^, Er^3+^ PQDs were identified, appearing at 980 nm and 1540 nm, assigned to Yb^3+^ (^2^F_5/2_ - ^2^F_7/2_) and Er^3+^ (^4^I_13/2_ - ^4^I_15/2_). Similarly to the previous reported, they originate from the quantum cutting energy transfer from CsPbCl_3_ PQDs to Yb^3+^, and further Yb^3+^ to Er^3+^^57,58^. Furthermore, the introduction of Ce^3+^ regards as an intermediate bridge to match the energy between CsPbCl_3_ PQDs and NIR quantum cutting emission of Yb^3+^^59^. Meanwhile, the PLQYs is estimated by^23^:

6 $${PLQYs}=\frac{{N}_{{em}}}{{N}_{{abs}}}=\frac{\int {I}_{{sample}}\left({\rm{\lambda }}\right)-{I}_{{ref}}\left({\rm{\lambda }}\right)d{\rm{\lambda }}}{\int {E}_{{ref}}\left({\rm{\lambda }}\right)-{E}_{{sample}}\left({\rm{\lambda }}\right)d{\rm{\lambda }}}$$

where *N*_*em*_ and *N*_*abs*_ are the number of emission and absorption photons of PQDs. *I*_*sample*_ and *E*_*sample*_ present the spectral intensity of the emitted light and excitation light of samples, and *I*_*ref*_ and *E*_*ref*_ are the spectral intensity of the emitted light and excitation light for a reference cuvette containing toluene. It should be highlighted that the PLQYs of CsPbCl_3_:Cr^3+^, Ce^3+^, Yb^3+^, Er^3+^ PQDs reaches to 179% (Fig. 5c). The higher PLQYs are concluded in the following three aspects: (1) reduced k_nr_ of CsPbCl_3_ PQDs after Cr^3+^ doping, (2) enhanced energy transfer from CsPbCl_3_ PQDs to Yb^3+^ and improved DUV absorption after Ce^3+^, (3) NIR quantum cutting emission (^2^F_5/2_ - ^2^F_7/2_) after Yb^3+^ doping. The energy transfer process for Cr^3+^, Ce^3+^, Yb^3+^, Er^3+^ doped CsPbCl_3_ PQDs was represented in Fig. 5d.

Then, the CsPbCl_3_:Cr^3+^, Ce^3+^, Yb^3+^, Er^3+^ PQDs were incorporated into a PMMA polymer matrix to form QC–LC. The performance of the QC–LC was achieved using an integrating sphere. As seen in Fig. 5e and S24, the QC–LC presents the similar emission spectra with CsPbCl_3_:Cr^3+^, Ce^3+^, Yb^3+^, Er^3+^ PQDs and high transparency. Considering directly integrating one of edge of QC–LC with the PDs, the emission intensity of the edge, face, and total of LC were recorded (Fig. 5e). The emission intensity of the edge is higher than that of face, attributed to the waveguide effect of QC–LC. The edge and face emission intensity ratio of the QC-LC was 73.61%, which is nearly to the ideal light trapping efficiency of 75%^13,60^. Moreover, the stability of CsPbCl_3_:Cr^3+^, Ce^3+^, Yb^3+^, Er^3+^ PQDs were also remarkably enhanced by incorporating them into LC, in which their PL intensity remains 97.5% after 100 days storage (Fig. 5f). Based on the high luminescence performance and stability and transparency, the QC–LC can be severed as a converter to improve the DUV–UV responsivity for broadband PDs.

**Fig. 6:**
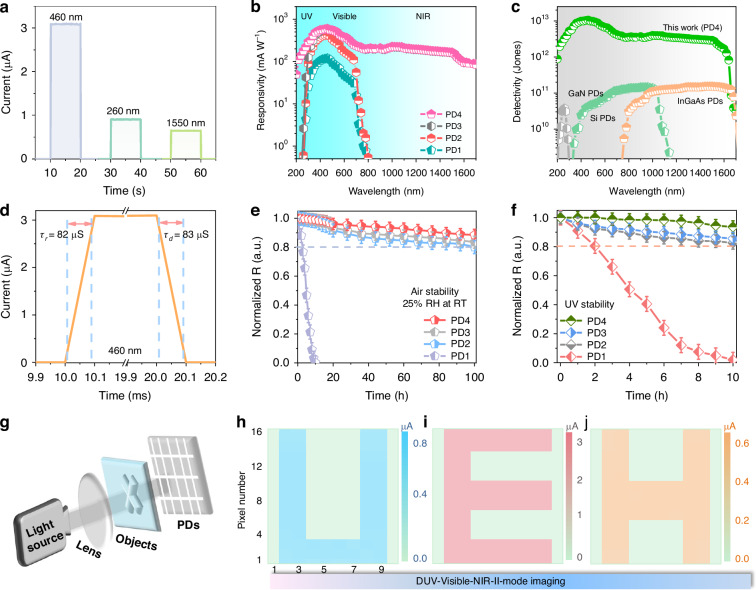
Performances of PDs. **a** *I*_p_–*t* curves of PD4 under the 260 nm, 460 nm, and 1550 nm illumination, respectively. **b** Responsivity of PD1–PD4. **c** Detectivity of this work (PD4), GaN PDs, Si PDs and InGaAs PDs, respectively. **d** Response time of the PD4 under 460 nm illumination. **e**, **f** The evolution of responsivity under 25% RH at room temperature and UV radiation. **g** Schematic diagram of imaging arrays. DUV–Visible-NIR-II mode imaging output for “U, E, H” mask under 260 nm (**h**), 460 nm (**i**) and 1550 nm (**j**), respectively

Figures 6a and S25 shows the photocurrent-time (*I*_p_–*t*) response curves of FTO / SnO_2_ / CsPbI_3_ PQDs / MoO_3_ / Au (PD1), FTO / SnO_2_ / CsPbI_3_:Ho^3+^ PQDs / MoO_3_ / Au (PD2), FTO / SnO_2_ / CsPbI_3_:Ho^3+^-PbS QDs heterojunction / MoO_3_ / Au (PD3), and QC-LC / FTO / SnO_2_ / CsPbI_3_:Ho^3+^-PbS QDs heterojunction / MoO_3_ / Au (PD4) in dark and 260 nm, 460 nm, and 1550 nm illumination at a bias of 0.5 V, respectively. It can be seen that the I_p_ reach to 3.09 µA of PD4, whereas only 0.84 µA for PD1 under 460 nm illumination. Compared with the PD1, the I_p_ of PD4 increase 3.68 folds for 460 nm, due to the reduce density of defects and boost carriers mobility after Ho^3+^ doping, and passivized surface vacancy by PbS QDs. Besides, the dark current (I_d_) of PD1–PD4 were recorded in Fig. S26. The value of I_d_ was about 2.44 pA for PD1, which decreases to 0.26 pA for PD4. Furthermore, the light to dark current ratio of PD4 was 1.19 × 10^7^, which further suggest the formation of uniform CsPbI_3_:Ho^3+^—PbS QDs heterojunction film^35,61^. Interestingly, the high I_p_ signals of DUV (0.91 μA of 260 nm) and NIR-II (0.64 μA of 1550 nm) were observed for PD4. The enhancement is mainly due to the strong absorption in the DUV region and extremely efficient NIR quantum cutting emission of QC–LC, and high performance NIR-II broadband absorption of PbS QDs. Figure 6b, c and S27–S28 shows the responsivity (R), detectivity (D*), and external quantum efficiency (EQE) of PD1–PD4 (Supplementary Note 6). Compared to the PD1 with lower R (119.04 mA W^-1^) and D* (5.43 × 10^11^ Jones) and EQE (16.9%), the maximum R and D* and EQE of the PD4 were calculated to be 596.62 mA W^-1^ and 1.05 × 10^13^ Jones and 84.6% under 460 nm illumination. Furthermore, these values of PD4 were largely enhanced in the DUV–UV and NIR-II region, realizing a broadband spectral response ranging from 200 nm to 1700 nm, where the R and D* and EQE of PD4 were 180.6 mA W^-1^ and 164.5 mA W^-1^, 3.19 × 10^12^ Jones and 2.23 × 10^12^ Jones, and 45.3% and 7.2% under 260 nm and 1550 nm, respectively. The statistical analysis on 25 devices for PD4 under 260 nm, 460 nm, and 1550 nm illumination, respectively, showing the excellent repeatability of this broadband PDs (Fig. S29). Importantly, the D* of PD4 improves two orders that of commercial GaN PDs and Si PDs and InGaAs PDs under the same detection condition. Meanwhile, the noise current (I_n_) of PD4 (2.34 × 10^-14 ^A Hz^-1/2^) was lower than that of PD1 (8.33 × 10^-14 ^A Hz^-1/2^) at 1 Hz in Fig. S30. According to the noise-equivalent power (NEP) equation, the D* can also be calculation by ref. ^6^:

7 $$D* =\frac{\sqrt{{SB}}}{{NEP}}({cm}{{Hz}}^{-\frac{1}{2}}{W}^{-1}{or\; Jones})$$

8 $${NEP}=\,\frac{{I}_{n}}{R}$$

where S and *B* are the active layer area and the electrical bandwidth. The D* of PD4 was obtained to be 7.71 × 10^11^ Jones of 260 nm, 2.55 × 10^12^ Jones of 460 nm, and 5.23 × 10^11^ Jones of 1550 nm, respectively (Fig. S31). Compared with the PD1, the D* of PD2–PD4 shows the consistent increases trend for it calculated by the dark current, indicating a great potential for weak light detection.

The photon-response times of PDs are exhibited in Fig. 6d and S33–S34. The rise (τ_r_) and decay times (τ_d_) of the PD4 were measured to be ~82 µs and ~83 µs under 460 nm illumination, much faster than that of the PD1 (~202 µs and ~223 µs). The fast response time can be attributed to the restrained defects after Ho^3+^ doping, and the enhanced charge extraction and transport by integrating with PbS QDs. Figure S35 represent the relative response trend versus the frequency of 460 nm light. The -3dB bandwidth of PD4 was calculated as 4.04 KHz, indicating a rapid response to 460 nm light signal. According to the law: *f*_*-3dB*_ = 0.35/*τ*_*r*_^62^ the response time of PD4 is estimated to be 86.6 µs, similar to the response time measured in Fig. 6d. Furthermore, compared to the recent works of broadband PDs (Table 1 and Fig. S36), this broadband PDs shows the higher D* and faster response time from DUV to NIR-II regions. As shown in Fig. S37, the R as a function of light power density (P) can be fitted by the formula: *R* *∝* *P*^*α*^^63^ where α were achieved to be 0.67, 0.56, and 0.66 for PD4 under 260 nm, 460 nm, and 1550 nm, respectively. The *α* value of the PD4 is not closer to ideal result (*α* = 1), suggesting that defects still exist in the heterojunction interface^64–66^. Figure 6e, f displays the normalized R variation curves of PD1–PD4 at humidity of 25% and under UV irradiation at room temperature. The R of the PD1 declines to 0% after 100 h storage and 10 h UV irradiation, while the R of the PD4 remained 89% and 99.3% for the original *R*-value after 100 h storage and 10 h UV irradiation. The enhanced air- and UV- stability of PDs would be attributed to the Ho^3+^ doping, PbS QDs hybridization, and the buffer layer of QC–LC in the devices.

**Table 1:** Recent works of broadband PDs

Materials	D* (Jones)	R_t_ (μs)	Wavelength (nm)	Ref.
PEDOT:PSS / PbS / CsPbCl_3_	5.78 × 10^10^	–	350–1100	^33^
CH_3_NH_3_PbI_3_ / C8BTBT	2.17 × 10^12^	7.1 × 10^4^	365–808	^67^
GeTe / WS_2_	1.67 × 10^11^	3 × 10^4^	450–980	^68^
PbS-SCN / CH_3_NH_3_PbI_3_	3.0 × 10^11^	4.2 × 10^4^	365–1550	^37^
PdSe_2_ / FA_1-x_Cs_x_PbI_3_	∼ 10^13^	3.5/4	200–1550	^35^
CH_3_NH_3_PbI_3_ / PbSe	1.2 × 10^8^	3 × 10^3^	300–1500	^61^
CsPbBr_3_ / PbS QDs	1.13 × 10^10^	11.5	400–1130	^69^
GQD-PEDOT: PSS / Si	8 × 10^11^	80/70	300–1100	^70^
FAPbI_3_	9.4 × 10^11^	6.1 × 10^4^	340–700	^71^
Gd_3_Fe_5_O_12_ / Gr / Gd_3_Fe_5_O_12_ / MoS_2_	4.504 × 10^12^	2.9 × 10^5^/4.9 × 10^5^	300–1500	^72^
CQDs-halide / CQDs-EDT	2.5 × 10^12^	7.79/11.48	400–1400	^73^
2D Bi_2_O_2_Se / CsBi_3_I_10_	1.22 × 10^12^	4.1/6.2	365–1500	^74^
p-WSe_2_ / n-Ge	2.1 × 10^10^	30	450–1550	^75^
Bi_2_Te_3_-SnSe-Bi_2_Te_3_	6 × 10^10^	–	370–808	^76^
FA_0.5_MA_0.45_Cs_0.05_Pb_0.5_Sn_0.5_I_3_	2.21 × 10^11^	0.043	300–1050	^6^
MoS_2_ / CdTe	6.1 × 10^10^	43.7/82.1	200–1700	^77^
FAMAPbI_3_	4.3 × 10^12^	6.3 × 10^4^	300–800	^78^
PEDOT: PSS / PbS / CsPbCl_3_	6.77 × 10^12^	6.5 × 10^5^	405–808	^36^
CH_3_NH_3_PbI_3_	∼10^11^	4.1/3.6	300–750	^79^
Cr/Ce/Mn-LC / CsPbI_3_:Er^3+^ / BHJ	2.46 × 10^12^	350/300	200–1000	^23^
QC-LC / FTO / SnO_2_ / CsPbI_3_:Ho^3+^-PbS QDs / MoO_3_ / Au	1.05×10^13^	82/83	200–1700	This work

Based on the outstanding DUV–Visible-NIR-II detection performance, the imaging application of this broadband PD was further explored. As presented in Fig. 6g, the imaging schematic is composed by different light source (DUV of 260 nm, Visible of 460 nm, and NIR-II of 1550 nm), lens, object letters (“U”, “E”, and “H”), broadband PDs and signal collector. We fabricated a 10 × 16 pixel detector array as the each photograph shown in Fig. 6h–j. The thickness of the detectors is 1 mm and the electrode area of each pixel is 1 × 1 mm^2^. It can be seen that the letters were clearly achieved with high light to dark current ratios under different illuminations, highlighting that the excellent performance of broadband detection and imaging capability for the QC–LC / FTO / SnO_2_ / CsPbI_3_:Ho^3+^-PbS QDs heterojunction / MoO_3_ / Au PDs. Moreover, these results strongly indicate that the remarkable adaptability of the PDs and great promising for broadband imaging without low temperature working conditions.

## Discussion

In this work, an excellent performance DUV–Visible-NIR-II broadband PD was successfully fabricated based on QC–LC and CsPbI_3_:Ho^3+^—PbS QDs heterojunction, exhibiting the high detectivity of 3.19 × 10^12^ Jones at 260 nm, 1.05 × 10^13^ Jones at 460 nm, and 2.23 × 10^12^ Jones at 1550 nm, respectively. The optimized CsPbI_3_:Ho^3+^ PQDs demonstrated low trap density and high charge mobility compared with the undoped CsPbI_3_ PQDs, enabling superb responsivity and detectivity of visible region. Then, the hybridization of CsPbI_3_: Ho^3+^—PbS QDs heterojunction successfully expand the response of broadband PDs to the NIR-II region, due to the narrow bandgap and large absorption coefficient of PbS QDs. Meanwhile, high efficiency NIR quantum cutting emission (PQLYs ~ 179%) and strong DUV–UV absorption of Cr^3+^, Ce^3+^, Yb^3+^, Er^3+^ doped CsPbCl_3_ PQDs were embedded to form QC–LC, which can be integrated with the FTO side to improve the responsivity of PDs for DUV–UV light. Our work shows a promising path for fabricated the high performancebroadband PDs and imaging capability.

## Materials and methods

### Materials

Cs_2_CO_3_ (99.9%), 1-octadecene (ODE, 90%), oleic acid (OA, 85%), oleylamine (OAm, 70%), PbO (99.99%), PbI_2_ (99.99%), HoI_3_ (99.99%), PbCl_2_ (99.9%), CrCl_3_ (99.99%), CeCl_3_ (99.99%), YbCl_3_ (99.99%), ErCl_3_ (99.99%), bis(trimethylsilyl) sulfide, toluene, cyclohexane and ethyl acetate (99%) were purchased from Mackline and were used without further purification.

### Synthesis of CsPbI_3_ PQDs

0.8 g Cs2CO3 was added into a mixture of 30 mL of ODE and OA (2.5 mL) and then heated to 150 °C until the white powder was completely dissolved. The mixture was then kept at 120 °C to obtained the Cs-oleate. Then, PbI2 (0.3 mmol), OAm (1.5 mL), OA (1.5 mL), and ODE (10 mL) were added to a 50-mL 3-neck round-bottomed flask and were evacuated with N2, by heating the solution to 120 °C for 1 h. The temperature of the solution was then increased to 180 °C for 10 min. Finally, the Cs-oleate (1 mL) was swiftly injected into the solution. After 10 s, the solution was cooled in an ice bath. The CsPbI_3_ PQDs were precipitated and then centrifuged, followed by dissolution in toluene.

### Synthesis of CsPbI_3_:Ho^3+^ PQDs

HoI_3_ (x mmol), PbI_2_ (0.3 mmol), OAm (1.5 mL), OA (1.5 mL), and ODE (10 mL) were adequately dissolved at 120 °Cfor 1 h under purging N_2_ gas. The following steps were the same with synthesis of CsPbI_3_ PQDs.

### Synthesis of Cr^3+^,Ce^3+^,Yb^3+^,Er^3+^ doped CsPbCl_3_ PQDs

For the CsPbCl3: Cr^3+^, Ce^3+^, Yb^3+^, Er^3+^ PQDs, PbCl2 (0.5 mmol), CrCl3 (0.3 mmol), CeCl_3_(0.2 mmol), YbCl3 (0.3 mmol), ErCl3 (0.2 mmol) were loaded into round-bottom flask with ODE (10 mL), OAm (1.5 mL) and OA (1.5 mL). The following steps were the same with the synthesis of the Ho^3+^ doped CsPbI_3_ PQDs.

### Fabrication of QC-LC

0.8 g PMMA (MW ~ 350000) was dispersed in 5 mL toluene by sonication, where toluene solution (3 mL) of CsPbCl3: Cr^3+^, Ce^3+^, Yb^3+^, Er^3+^ PQDs were added. The mixture was sealed and stirred overnight to obtain homogenous slurry. The slurry was centrifuged at 2000 rpm and the supernatants were used for LC fabrication. The above supernatants were spin-coating onto borosilicate glass substrates.

### Syntheses of PbS QDs

Firstly, PbO (0.36 g), OA (1 mL), and ODE (15 mL) were stirred and heated to 145 °C under nitrogen atmosphere. Then, ODE (4.0 mL) containing bis (trimethylsilyl) sulfide (168 µL) was quickly injected into the reaction flask, and meanwhile the heater was switched off. The hot solution was naturally cooled down, and the formed oil-soluble PbS QDs were precipitated. The purification was carried by isopropyl alcohol and acetone. Finally, the PbS CQDs were dispersed in toluene to produce a 50 mg mL^-1^ solution.

### Preparation of the CsPbI_3_:Ho^3+^-PbS QDs heterojunction composite

In a typical procedure, a 200 μL CsPbI_3_:Ho^3+^ solution (10 mg mL^-1^, toluene) was mixed with 2 mL of PbS solution (1 mg mL^-1^, toluene), and the total volume remained at 5 mL. Under dark conditions, the suspension was ultrasonicated for 10 min and stirred for 0.5 h. Finally, the CsPbI_3_:Ho^3+^—PbS CQDs heterojunction composites were collected by centrifuging at 8000 rpm for 5 min.

### Device fabrication

FTO coated glass substrates were etched by zinc powder and HCl to define the electrode patterns and washed in deionized water, acetone, and ethanol for 20 min, respectively. The ultraviolet-ozone was used to remove the organic residues of FTO surface. To fabricate the compact SnO_2_ layer, the SnO_2_ colloid solution by water to the concentration of 2.14 wt% and was spin-coated on FTO substrates at 5000 rpm for 30 s and then annealed at 150 °C for 30 min. The CsPbI_3_:Ho^3+^—PbS CQDs heterojunction film was fabricated on the SnO_2_ layer by spin-coating at 2000 rpm for 40 s. For broadband PDs, MoO3 and Au was evaporated on CsPbI_3_:Ho^3+^—PbS CQD film layer. Then, the edge surface of QC–LC with the edge size of 1 × 0.4 mm^2^ was attached and fixed to the ITO layer of PD with area of 1 × 1 mm^2^. When UV light radiation on the face of QC–LC, then the emitted 400–1700 nm light is coupled out of the edge surface into the FTO of PDs. The visible and NIR lights directly pass through the FTO and reach the PD. Because the QC–LC only occupies a part of surface of the FTO layer and has high transparency for photons with the longer wavelength (>400nm), which would not affect the light collection of PDs.

### Characterization

UV/vis-NIR absorption spectra were measured with a Shimadzu UV-3600PCscanning spectrophotometer in the range from 200 to 2500 nm. Patterns were recorded in thin film mode on a Bruker AXS D8 diffractmeter by Cu Kαradiation (*λ* = 1.54178 Å). Atomic Force Microscope was texted using a DI Innova AFM (Bruker) in light tapping mode. The morphology of the products was recorded with a Hitachi H-8100IV transmission electron microscope (TEM) under an acceleration voltage of 200 kV. A Visible-NIR photomultiplier combined with a double-grating monochromator were used for spectral collection. The X-ray photoelectron spectroscopy (XPS) was carried out in a Kratos Axis Ultra DLD spectrometer equipped with a monochromatic Al Kα X-ray source (*hν* = 1486.6 eV) operated at 150 W with a multichannel plate, and a delay line detector under 1.0 × 10^-9 ^Torr vacuum. Nanosecond fluorescence lifetime experiments were performed by the time correlated single-photon counting system. The Mott–Schottky curves via capacitance-voltage measurements of CsPbI_3_:Ho^3+^ PQDs were obtained by a Princeton electrochemical workstation. The Xe lamp with the spectral range from 200 nm to 2500 nm equipped with a monochromator was used to generate the monochromatic light to conduct the spectral response measurements. Actually, the intensity of Xe lamp is weak in the region of 200 nm–300 nm, thus we must correct it before the measurement.

## Supplementary information

Supplementary information

## References

[1] Guo, Z. N. et al. In-situ neutron-transmutation for substitutional doping in 2D layered indium selenide based phototransistor. *eLight* **2**, 9 (2022).

[2] Dong, H. Metal Halide Perovskite for next-generation optoelectronics: progresses and prospects. *eLight* **3**, 3 (2023).

[3] Wang, P. S. et al. Van der Waals two-color infrared photodetector. *Light Sci. Appl.* **11**, 6 (2022). 34974520 10.1038/s41377-021-00694-4 PMC8720310

[4] Ji, Y. N. et al. Semiconductor plasmon enhanced monolayer upconversion nanoparticles for high performance narrowband near-infrared photodetection. *Nano Energy* **61**, 211–220 (2019).

[5] Chen, X. X. et al. Ultrasensitive broadband position-sensitive detector based on graphitic carbon nitride. *Nano Res.* **16**, 1277–1285 (2023).

[6] Ma, N. N. et al. Stable and sensitive tin-lead perovskite photodetectors enabled by azobenzene derivative for near-infrared acousto-optic conversion communications. *Nano Energy* **86**, 106113 (2021).

[7] Hu, W. et al. Germanium/perovskite heterostructure for high-performance and broadband photodetector from visible to infrared telecommunication band. *Light Sci. Appl.* **8**, 106 (2019). 31798845 10.1038/s41377-019-0218-y PMC6872564

[8] Zhang, M. J. et al. Perovskite quantum dots embedded composite films enhancing UV response of silicon photodetectors for broadband and solar-blind light detection. *Adv. Opt. Mater.* **6**, 1800077 (2018).

[9] Cao, G. Q. et al. Multicolor broadband and fast photodetector based on InGaAs-insulator-graphene hybrid heterostructure. *Adv. Electron. Mater.* **6**, 1901007 (2020).

[10] Khan, J. et al. Tuning the surface-passivating ligand anchoring position enables phase robustness in CsPbI_3_ perovskite quantum dot solar cells. *ACS Energy Lett.* **5**, 3322–3329 (2020).

[11] Wang, Y. et al. Surface ligand management aided by a secondary amine enables increased synthesis yield of CsPbI_3_ perovskite quantum dots and high photovoltaic performance. *Adv. Mater.* **32**, 2000449 (2020). 10.1002/adma.202000449 32609406

[12] Ding, N. et al. Upconversion ladder enabled super-sensitive narrowband near-infrared photodetectors based on rare earth doped florine perovskite nanocrystals. *Nano Energy* **76**, 105103 (2020).

[13] Luo, X. et al. Quantum-cutting luminescent solar concentrators using ytterbium-doped perovskite nanocrystals. *Nano Lett.* **19**, 338–341 (2019). 30525678 10.1021/acs.nanolett.8b03966

[14] Yang, G. et al. Efficient quantum cutting of lanthanum and ytterbium ions co-doped perovskite quantum dots towards improving the ultraviolet response of silicon-based photodetectors. *J. Alloy. Comp.* **921**, 166097 (2022).

[15] Xu, W. et al. Atomic-scale imaging of ytterbium ions in lead halide perovskites. *Sci. Adv.* **9**, eadi7931 (2023). 37656785 10.1126/sciadv.adi7931 PMC10854428

[16] Liu, K. K. et al. Water-induced MAPbBr_3_@PbBr(OH) with enhanced luminescence and stability. *Light Sci. Appl.* **9**, 44 (2020). 32194958 10.1038/s41377-020-0283-2 PMC7078192

[17] Bi, C. H. et al. Improved stability and photodetector performance of CsPbI_3_ perovskite quantum dots by ligand exchange with aminoethanethiol. *Adv. Funct. Mater.* **29**, 1902446 (2019).

[18] Zhao, C. et al. Polymer-assisted phase stable γ-CsPbI_3_ perovskite film for self-powered and ultrafast photodiodes. *Adv. Mater. Interf.* **9**, 2102212 (2022).

[19] Kim, H. et al. Bias-modulated multicolor discrimination enabled by an organic-inorganic hybrid perovskite photodetector with a p-i-n-i-p configuration. *Laser Photon. Rev.* **14**, 2000305 (2020).

[20] Liu, K. L. et al. Highly efficient and stable red perovskite quantum dots through encapsulation and sensitization of porous CaF_2_: Ce, Tb nanoarchitectures. *Nanoscale* **14**, 4263–4270 (2022). 35244135 10.1039/d2nr00544a

[21] Shen, X. Y. et al. Zn-alloyed CsPbI_3_ nanocrystals for highly efficient perovskite light-emitting devices. *Nano Lett.* **19**, 1552–1559 (2019). 30741555 10.1021/acs.nanolett.8b04339

[22] Shao, L. et al. Near-infrared-pumped photon upconversion in CsPbI_3_ and CaF_2_: Yb^3+^/Ho^3+^ nanocomposites for bio-imaging application. *Mater. Today Phys.* **21**, 100495 (2021).

[23] Ding, N. et al. A novel approach for designing efficient broadband photodetectors expanding from deep ultraviolet to near infrared. *Light Sci. Appl.* **11**, 91 (2022). 35410451 10.1038/s41377-022-00777-w PMC9001727

[24] Sulaman, M. et al. Hybrid bulk-heterojunction of colloidal quantum dots and mixed-halide perovskite nanocrystals for high-performance self-powered broadband photodetectors. *Adv. Funct. Mater.* **32**, 2201527 (2022).

[25] Ding, N. et al. Europium-doped lead-free Cs_3_Bi_2_Br_9_ perovskite quantum dots and ultrasensitive Cu^2+^ detection. *ACS Sustain. Chem. Eng.* **7**, 8397–8404 (2019).

[26] Zhong, Y. et al. Circularly polarized luminescence of lanthanide complexes: from isolated individuals, discrete oligomers, to hierarchical assemblies. *InfoMat* **5**, e12392 (2023).

[27] Jin, S. L. et al. Compact ultrabroadband light-emitting diodes based on lanthanide-doped lead-free double perovskites. *Light Sci. Appl.* **11**, 52 (2022). 35256583 10.1038/s41377-022-00739-2 PMC8901751

[28] Zhang, G. D. et al. Er^3+^/Yb^3+^-based halide double perovskites with highly efficient and wide ranging antithermal quenching photoluminescence behavior for light-emitting diode applications. *Laser Photon. Rev.* **16**, 2200078 (2022).

[29] Ding, N. et al. Highly-sensitive, stable, and fast-response lead-free Cs_2_AgBiBr_6_ double perovskite photodetectors enabled by synergistic engineering of doping Na^+^/Ce^3+^ and integrating Ag nanoparticles film. *Laser Photon. Rev.* **16**, 2200301 (2022).

[30] Wang, L. et al. Exploration of nontoxic Cs_3_CeBr_6_ for violet light-emitting diodes. *ACS Energy Lett.* **6**, 4245–4254 (2021).

[31] Chiba, T. et al. Neodymium chloride-doped perovskite nanocrystals for efficient blue light-emitting devices. *ACS Appl. Mater. Interf.* **12**, 53891–53898 (2020). 10.1021/acsami.0c11736 33210903

[32] Zi, L. et al. X-ray quantum cutting scintillator based on CsPbCl_*x*_Br_3−*x*_: Yb^3+^ single crystals. *Laser Photon. Rev.* **17**, 2200852 (2023).

[33] Zhao, X. H. et al. Vertically stacked PEDOT: PSS/PbS/CsPbCl_3_ for flexible optoelectronic devices. *J. Alloy Comp.* **866**, 158997 (2021).

[34] Li, K. et al. Vertically stacked Au/PbS/CsPbCl_3_ phototransistors for plasmon-enhanced high-performance broadband photodetection. *ACS Appl. Electron. Mater.* **2**, 4080–4086 (2020).

[35] Zeng, L. H. et al. Multilayered PdSe_2_/perovskite Schottky junction for fast, self-powered, polarization-sensitive, broadband photodetectors, and image sensor application. *Adv. Sci.* **6**, 1901134 (2019). 10.1002/advs.201901134 PMC6774060 31592422

[36] Zhao, X. H. et al. Enhanced photodetection of perovskite nanoplatelet devices by vertically stacked PEDOT: PSS/PbS/CsPbCl_3_ architecture. *Mater. Lett.* **290**, 129467 (2021).

[37] Zhang, J. Y. et al. Toward broadband imaging: surface-engineered PbS quantum dot/perovskite composite integrated ultrasensitive photodetectors. *ACS Appl. Mater. Interf.* **11**, 44430–44437 (2019). 10.1021/acsami.9b14645 31680508

[38] Liu, Q. B. et al. Epitaxial growth of CsPbBr_3_-PbS vertical and lateral heterostructures for visible to infrared broadband photodetection. *Nano Res.* **14**, 3879–3885 (2021).

[39] Kang, C. H. et al. High-speed colour-converting photodetector with all-inorganic CsPbBr_3_ perovskite nanocrystals for ultraviolet light communication. *Light Sci. Appl.* **8**, 94 (2019). 31645937 10.1038/s41377-019-0204-4 PMC6804731

[40] Wu, Y. J. et al. Toward broad spectral response inverted perovskite solar cells: insulating quantum-cutting perovskite nanophosphors and multifunctional ternary organic bulk-heterojunction. *Adv. Energy Mater.* **12**, 2200005 (2022).

[41] Lyu, J. K. et al. Ni^2+^ and Pr^3+^ Co-doped CsPbCl_3_ perovskite quantum dots with efficient infrared emission at 1300 nm. *Nanoscale* **13**, 16598–16607 (2021). 34585206 10.1039/d1nr04455a

[42] Li, D. Y. et al. Cerium-doped perovskite nanocrystals for extremely high-performance deep-ultraviolet photoelectric detection. *Adv. Opt. Mater.* **9**, 2100423 (2021).

[43] Sun, R. et al. In situ preparation of two-dimensional ytterbium ions doped all-inorganic perovskite nanosheets for high-performance visual dual-bands photodetectors. *Nano Energy* **93**, 106815 (2022).

[44] Li, S. et al. Sodium doping-enhanced emission efficiency and stability of CsPbBr_3_ nanocrystals for white light-emitting devices. *Chem. Materials* **31**, 3917–3928 (2019).

[45] Sun, R. et al. Efficient single-component white light emitting diodes enabled by lanthanide ions doped lead halide perovskites via controlling Forster energy transfer and specific defect clearance. *Light Sci. Appl.* **11**, 340 (2022). 36470864 10.1038/s41377-022-01027-9 PMC9722690

[46] Hao, M. Y. et al. Dy^3+^ doped all-inorganic perovskite nanocrystals glass toward high-performance and high-stability silicon photodetectors. *Laser Photon. Rev.* **17**, 2200748 (2023).

[47] Pan, G. C. et al. Doping lanthanide into perovskite nanocrystals: highly improved and expanded optical properties. *Nano Lett.* **17**, 8005–8011 (2017). 29182877 10.1021/acs.nanolett.7b04575

[48] Ding, J. et al. Eu^3+^ doped CsPbCl_2_Br_1_ nanocrystals glass for enhanced the ultraviolet response of Si photodetectors. *J. Lumin.* **254**, 119530 (2023).

[49] Protesescu, L. et al. Nanocrystals of cesium lead halide perovskites (CsPbX_3_, X = Cl, Br, and I): novel optoelectronic materials showing bright emission with wide color gamut. *Nano Lett.* **15**, 3692–3696 (2015). 25633588 10.1021/nl5048779 PMC4462997

[50] Wang, K. et al. Efficient perovskite solar cells by hybrid perovskites incorporated with heterovalent neodymium cations. *Nano Energy* **61**, 352–360 (2019).

[51] Liu, J. et al. Flexible and broadband colloidal quantum dots photodiode array for pixel-level X-ray to near-infrared image fusion. *Nat. Commun.* **14**, 5352 (2023). 37660051 10.1038/s41467-023-40620-3 PMC10475073

[52] Cao, Y. H. et al. Asymmetric strain-introduced interface effect on the electronic and optical properties of the CsPbI_3_/SnS van der Waals heterostructure. *Adv. Mater. Interf.* **6**, 1901330 (2019).

[53] Liu, B. et al. Two-dimensional van der Waals heterostructures constructed via perovskite (C_4_H_9_NH_3_)_2_XBr_4_ and black phosphorus. *J. Phys. Chem. Lett.* **9**, 4822–4827 (2018). 30091614 10.1021/acs.jpclett.8b02078

[54] Volonakis, G. & Giustino, F. Ferroelectric graphene–perovskite interfaces. *J. Phys. Chem. Lett.* **6**, 2496–2502 (2015). 26266725 10.1021/acs.jpclett.5b01099

[55] Ding, N. et al. Extremely efficient quantum-cutting Cr^3+^, Ce^3+^, Yb^3+^ tridoped perovskite quantum dots for highly enhancing the ultraviolet response of silicon photodetectors with external quantum efficiency exceeding 70. *Nano Energy* **78**, 105278 (2020).

[56] Zhao, J. et al. Efficient dual-mode emissions of high-concentration erbium ions doped lead-free halide double perovskite single crystals. *J Alloy Comp.* **895**, 162601 (2022).

[57] Milstein, T. J., Kroupa, D. M. & Gamelin, D. R. Picosecond quantum cutting generates photoluminescence quantum yields over 100% in ytterbium-doped CsPbCl_3_ nanocrystals. *Nano Letters* **18**, 3792–3799 (2018). 29746137 10.1021/acs.nanolett.8b01066

[58] Zhu, Y. S. et al. Effective infrared emission of erbium ions doped inorganic lead halide perovskite quantum dots by sensitization of ytterbium ions. *J. Alloy Comp.* **835**, 155390 (2020).

[59] Zhou, D. L. et al. Cerium and ytterbium codoped halide perovskite quantum dots: a novel and efficient downconverter for improving the performance of silicon solar cells. *Adv. Mater.* **29**, 1704149 (2017). 10.1002/adma.201704149 28961346

[60] Wu, K. F., Li, H. B. & Klimov, V. I. Tandem luminescent solar concentrators based on engineered quantum dots. *Nature Photon.* **12**, 105–110 (2018).

[61] Yu, Y. et al. Broadband phototransistor based on CH_3_NH_3_PbI_3_ perovskite and PbSe quantum dot heterojunction. *J. Phys. Chem. Lett.* **8**, 445–451 (2017). 28050910 10.1021/acs.jpclett.6b02423

[62] He, M. et al. Sn-based self-powered ultrafast perovskite photodetectors with highly crystalline order for flexible imaging applications. *Adv. Funct. Mater.* **33**, 2300282 (2023).

[63] Gong, M. G. et al. High-performance all-inorganic CsPbCl_3_ perovskite nanocrystal photodetectors with superior stability. *ACS Nano* **13**, 1772–1783 (2019). 30689349 10.1021/acsnano.8b07850

[64] Dubey, A. et al. Aluminum plasmonics enriched ultraviolet GaN photodetector with ultrahigh responsivity, detectivity, and broad bandwidth. *Adv. Sci.* **7**, 2002274 (2020). 10.1002/advs.202002274 PMC7740085 33344129

[65] Wang, F. K. et al. 2D metal chalcogenides for IR photodetection. *Small* **15**, 1901347 (2019). 10.1002/smll.201901347 31111680

[66] Kim, H. et al. Solution-processed phototransistors combining organic absorber and charge transporting oxide for visible to infrared light detection. *ACS Appl. Mater. Interf.* **11**, 36880–36885 (2019). 10.1021/acsami.9b08622 31524369

[67] Xia, H. Y. et al. Flexible and air-stable perovskite network photodetectors based on CH_3_NH_3_PbI_3_/C8BTBT bulk heterojunction. *Appl. Phys. Lett.* **112**, 233301 (2018).

[68] Qu, J. Y. et al. Space-confined growth of ultrathin P-type GeTe nanosheets for broadband photodetectors. *Small*. **20**, 2309391 (2024). 10.1002/smll.202309391 38456381

[69] Zhao, H. L. et al. Self-driven visible-near infrared photodetector with vertical CsPbBr_3_/PbS quantum dots heterojunction structure. *Nanotechnology* **31**, 035202 (2020). 31585442 10.1088/1361-6528/ab4b17

[70] Tsai, M. L. et al. Omnidirectional harvesting of weak light using a graphene quantum dot-modified organic/silicon hybrid device. *ACS Nano* **11**, 4564–4570 (2017). 28430415 10.1021/acsnano.6b08567

[71] Wang, M. et al. Moisture-triggered self-healing flexible perovskite photodetectors with excellent mechanical stability. *Adv. Mater.* **33**, 2100625 (2021). 10.1002/adma.202100625 33734512

[72] Zhang, Z. et al. High-performance broadband flexible photodetector based on Gd_3_Fe_5_O_12_-assisted double van der Waals heterojunctions. *Microsyst. Nanoeng.* **9**, 84 (2023). 37408537 10.1038/s41378-023-00548-6 PMC10318041

[73] Liu, P. L. et al. Double-ended passivator enables dark-current-suppressed colloidal quantum dot photodiodes for CMOS-integrated infrared imagers. *InfoMat* **6**, e12497 (2024).

[74] Dang, L. Y. et al. Efficient carrier transport in 2D Bi_2_O_2_Se/CsBi_3_I_10_ perovskite heterojunction enables highly-sensitive broadband photodetection. *Small* **20**, 2306600 (2024). 10.1002/smll.202306600 38009782

[75] Lee, C. H. et al. Design of p-WSe_2_/n-Ge heterojunctions for high-speed broadband photodetectors. *Adv. Funct. Mater.* **32**, 2107992 (2022).

[76] Yao, J. D., Zheng, Z. Q. & Yang, G. W. All-layered 2D optoelectronics: a high-performance UV-vis-NIR broadband SnSe photodetector with Bi2Te3 topological insulator electrodes. *Adv. Funct. Mater.* **27**, 1701823 (2017).

[77] Wang, Y. G. et al. A room-temperature near-infrared photodetector based on a MoS_2_/CdTe p-n heterojunction with a broadband response up to 1700 nm. *J. Mater. Chem. C* **6**, 4861–4865 (2018).

[78] Wu, J. J. et al. Constructing high-performance solar cells and photodetectors with a doping-free polythiophene hole transport material. *Adv. Funct. Mater.* **34**, 2308584 (2024).

[79] He, J. et al. Improving photoelectric conversion with broadband perovskite metasurface. *Nano Lett.* **22**, 6655–6663 (2022). 35925801 10.1021/acs.nanolett.2c01979

