# Supplementary material for: Highly DUV to NIR-II responsive broadband quantum dots heterojunction photodetectors by integrating quantum cutting luminescent concentrators
Source: Light Sci Appl. 2024 Oct 15;13:289. doi: 10.1038/s41377-024-01604-0 (PMC11473808; doi:10.1038/s41377-024-01604-0)
Supplement: Supplementary file 1 — Supplementary information [file 41377_2024_1604_MOESM1_ESM.docx]

**Supplementary Information for：**

**Highly DUV to NIR-II responsive broadband quantum dots heterojunction photodetectors by integrating quantum cutting luminescent concentrators**

*Nan Ding, Wen Xu,* Hailong Liu, Yuhan Jing, Zewen Wang, Yanan Ji, Jinlei Wu, Long Shao, Ge Zhu,* Bin Dong**

*Key Laboratory of New Energy and Rare Earth Resource Utilization of State Ethnic Affairs Commission, School of Physics and Materials Engineering, Dalian Minzu University, 18 Liaohe West Road, Dalian 116600, China.*

*^*^E-mail: Prof. Wen Xu (xuwen@dlnu.edu.cn), Prof. Ge Zhu (zhuge@dlnu.edu.cn), Prof. Bin Dong (dong@dlnu.edu.cn）*

**Supplementary Note 1**

The increase of the band gap of PQDs could be divided into two reasons: (1) the quantum confinement effect; (2) the decrease of the lattice constant. The contribution of the quantum confinement effect is calculated as follows:^[1]^

*ΔΕ =* $\frac{\hbar^{2}{}^{2}}{{2m}_{r}R^{2}}$ $\frac{{1.786e}^{2}}{4_{0}R}$

where *R* represent the particle radius, *m_r_* denotes the effective mass of the exciton, *ε_0_* and *ε* are the vacuum permittivity and the relative dielectric constant of CsPbI_3_ bulk material. According to the equation, the calculated blue-shift of the band gap for PQDs within 12.6 meV with the particle size decrease from 11.6 nm to 10.5 nm. Compared to the experimental results, the decrease of lattice constant of PQDs after doping should be dominated.

**Supplementary Note 2**

The conductivity (*σ* ) of PQDs are obtained in FTO / PQDs /Ag devices. They can be estimated as follows:^[2]^

*I = σAD^-1^V*

where *A* is the contact area, *D* is the thickness of the PQDs films, *V* represents the applied voltage.

**Supplementary Note 3**

The Mott-Schottky curves via capacitance-voltage measurements of PQDs are obtained in FTO / SnO_2_ / PQDs /Ag devices. They can be estimated as follows:^[3]^

$\frac{1}{C^{2}}$ *=* $\frac{2}{A^{2}{}_{0}eN}\left( V_{b}-V \right)$

where *C* is the measured capacitance, *A* is the active area, *V* is the bias, *ε* is the static permittivity, *ε_0_* is the permittivity of free space, *e* is the elementary charge, and *N* is the doping density of the donor that represents carrier density in PDs.

**Supplementary Note 4**

The defect density (*N_t_*) and the carrier mobility (*μ*) of PQDs are obtained by dark current-voltage measurement technique in FTO / SnO_2_ / PQDs /Ag devices. They can be estimated as follows:^[4]^

$N_{t}$ *=* $\frac{2_{0}V_{TFL}}{{eL}^{2}}$

*μ =* $\frac{{8JL}^{3}}{9{}_{0}V^{2}}$

where *V_TFL_* is the trap-filled limit voltage,$L$ is the thickness of the PQDs film, *ε_0_* and *ε* are the vacuum permittivity and the relative dielectric constant of CsPbI_3_ bulk material, *J* and *V* are the current density and the applied voltage.

**Supplementary Note 5**

The density functional theory (DFT) calculations were carried out with the VASP code. The Perdew-Burke-Ernzerhof (PBE) functional within generalized gradient approximation (GGA) was used to process the exchange–correlation, while the projectoraugmented-wave pseudopotential (PAW) was applied with a kinetic energy cut-off of 500 eV, which was utilized to describe the expansion of the electronic eigenfunctions. The vacuum thickness was set to be 20 Å to minimize interlayer interactions. The Brillouin-zone integration was sampled by a Γ-centered 10 × 10 × 1 Monkhorst–Pack k-point. All atomic positions were fully relaxed until energy and force reached a tolerance of 1 × 10^-5^ eV and 0.03 eV Å^-1^, respectively. The dispersion corrected DFT-D method was employed to consider the long-range interactions.

**Supplementary Note 6**

These parameters satisfy the following equations:^[5]^

*R =* $\frac{I_{ph}-I_{d}}{PS}$

*D* =*$\sqrt{\frac{S}{{2eI}_{d}}}$*R*

*EQE = R*$\frac{hc}{e}$

where $I_{ph}$and $I_{d}$ are the photocurrent under the illumination of light and in the dark, $P$ and $S$ are the input light power density and the effective area (1×1 mm^2^), $h$ and $c$ are the Planck’s constant and the speed of light, and $e$ are the incident light wavelength and the elementary charge.


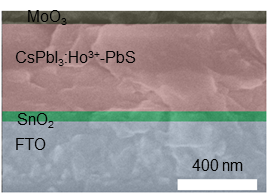


**Figure S1.** Cross-sectional SEM image of the broadband PDs.


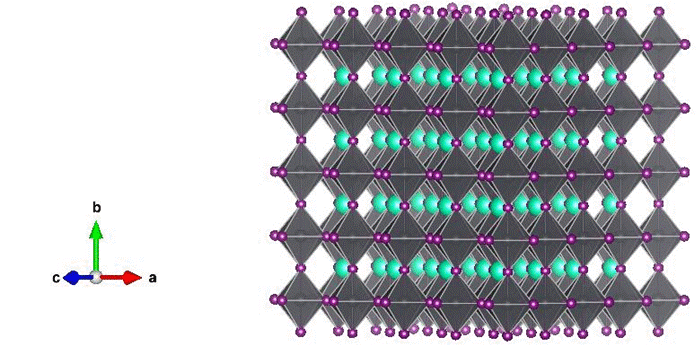


**Figure S2.** Structure diagram of CsPbI_3_ PQDs.


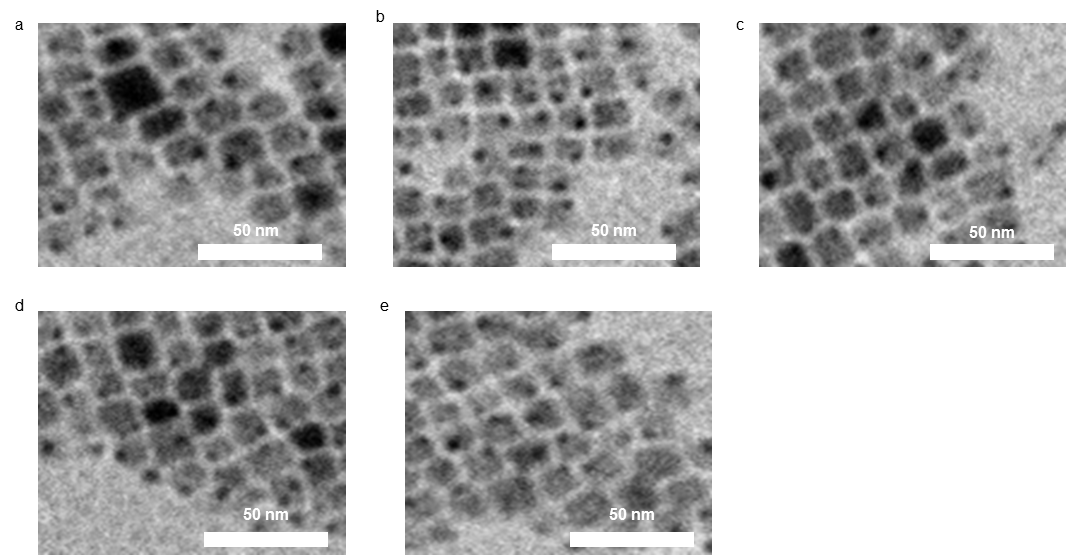


**Figure S3.** TEM images of undoped CsPbI_3_ and Ho^3+^ doped CsPbI_3_ PQDs with different doping concentrations (a-e: 0 %, 1.4 %, 3.2 %, 6.4 % and 8.3 %).


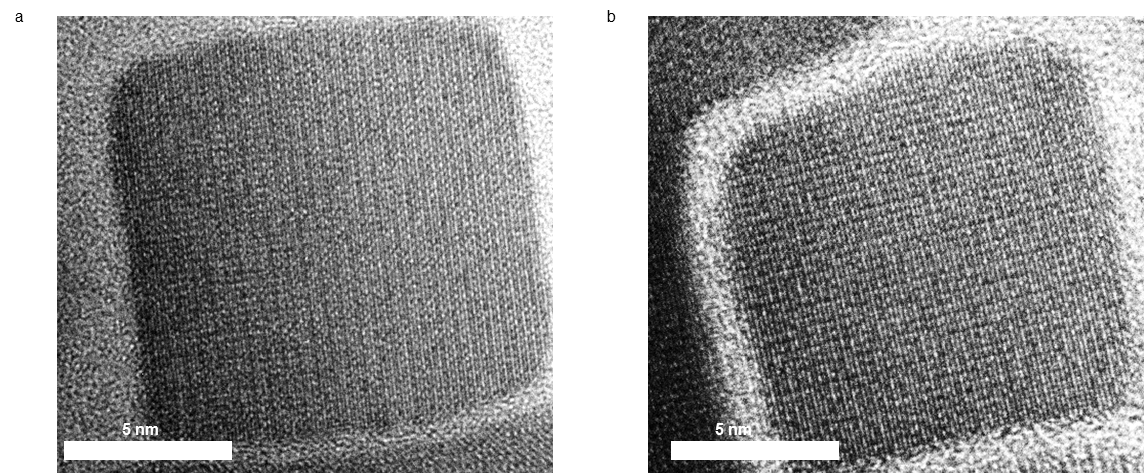


**Figure S4.** HR-TEM of undoped CsPbI_3_ (a) and CsPbI_3_:Ho^3+^ (6.4 %) PQDs (b).


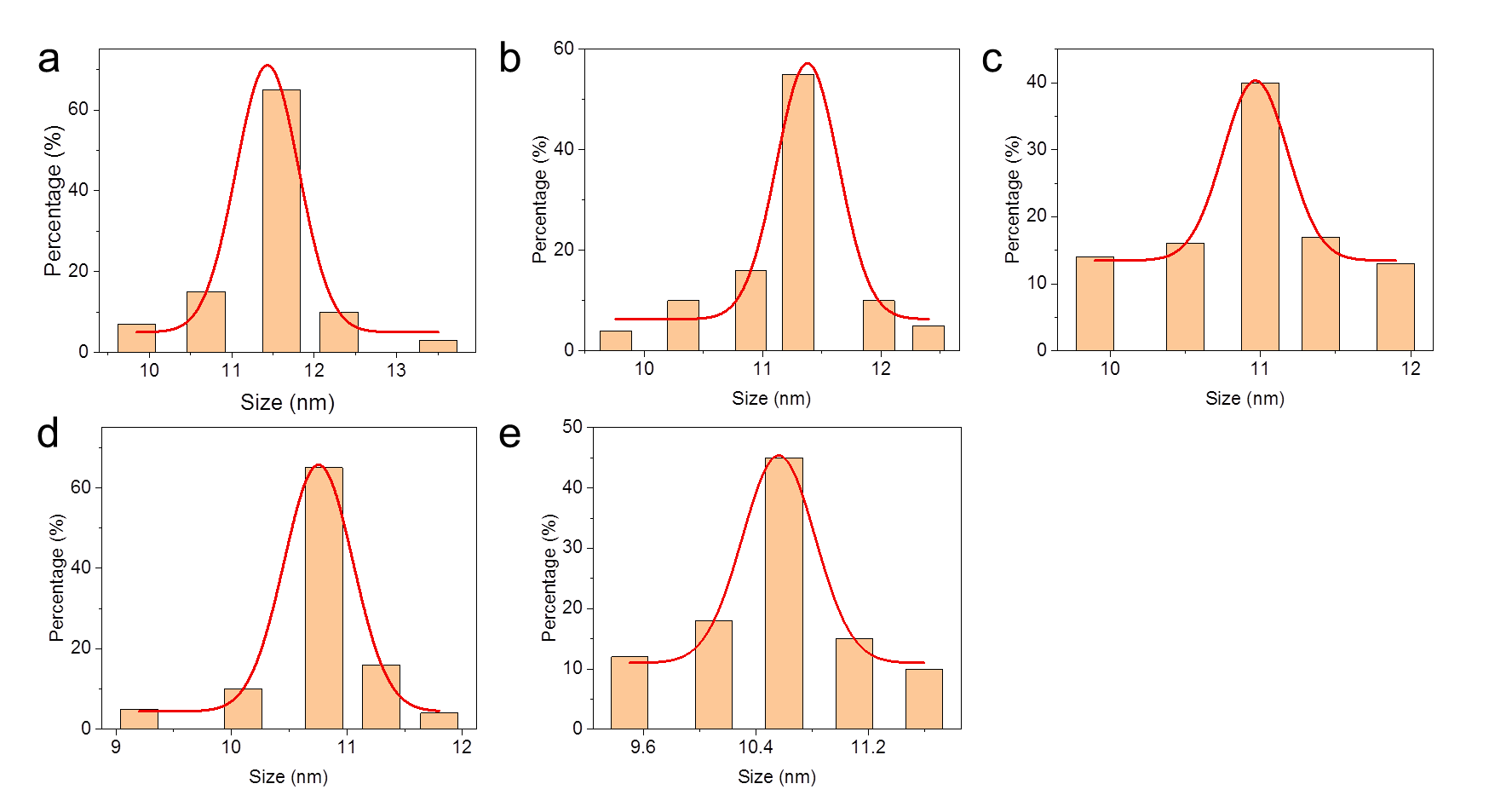


**Figure S5.** Size of undoped CsPbI_3_ and Ho^3+^ doped CsPbI_3_ PQDs with different doping concentrations (a-e: 0 %, 1.4 %, 3.2 %, 6.4 % and 8.3 %).


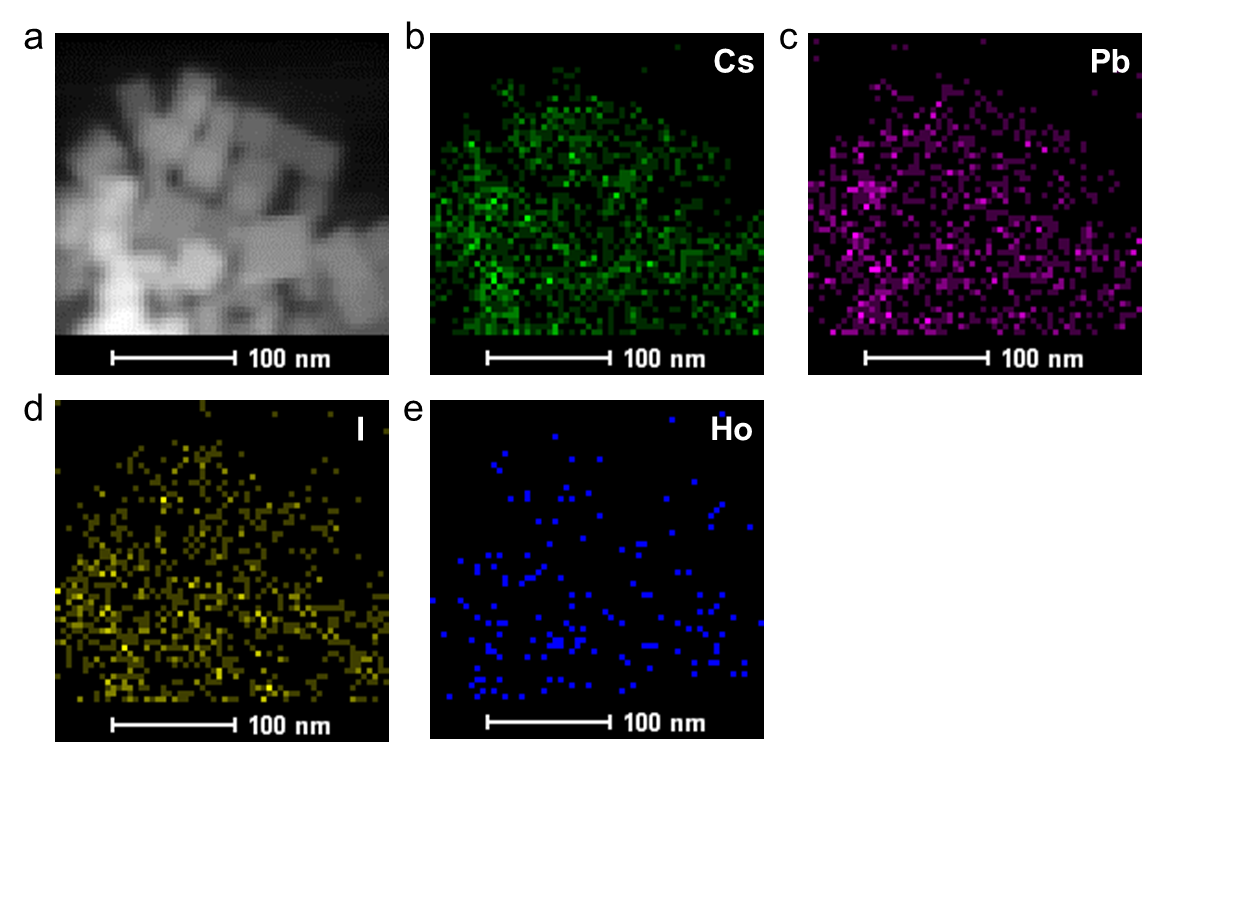


**Figure S6.** Mapping images of CsPbI_3_:Ho^3+^ (6.4 %) PQDs.


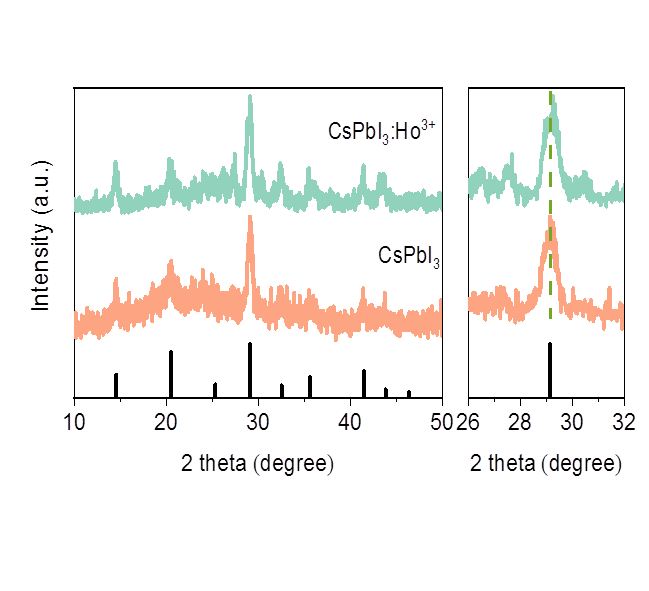


**Figure S7.** XRD patterns of CsPbI_3_ and CsPbI_3_:Ho^3+^ (6.4 %) PQDs.


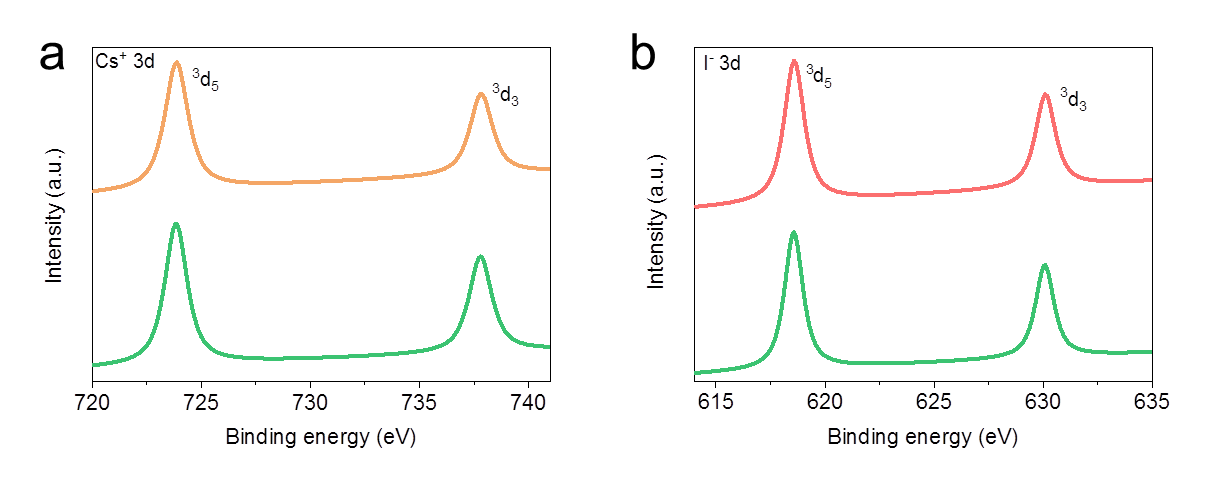


**Figure S8.** (a-b) HR-XPS spectra of Cs^+^ (3 *d*) and I^-^ (3 *d*) of CsPbI_3_ (bottom) and CsPbI_3_:Ho^3+^ (6.4 %) (top) PQDs.


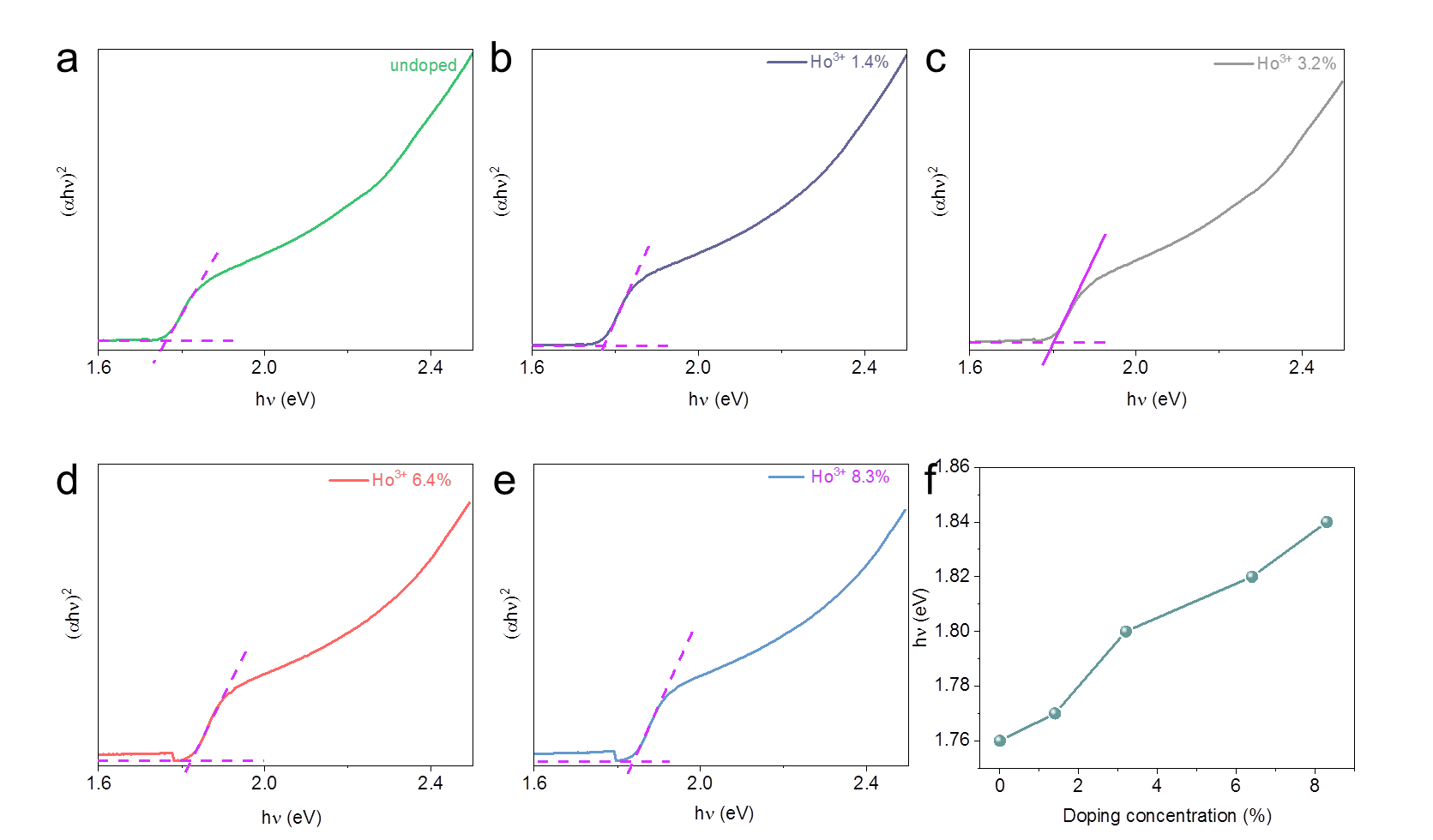


**Figure S9.** The exciton bandgaps of undoped CsPbI_3_ and Ho^3+^ doped CsPbI_3_ PQDs with different doping concentrations (a-e: 0 %, 1.4 %, 3.2 %, 6.4 % and 8.3 %).


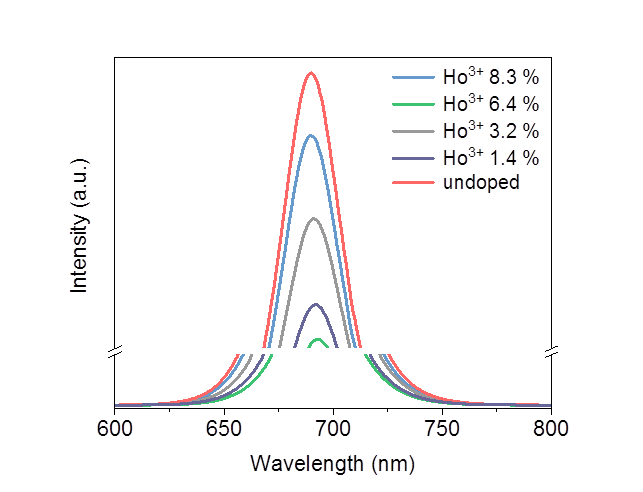


**Figure S10.** Emission spectra of CsPbI_3_ and CsPbI_3_:Ho^3+^ PQDs with different Ho^3+^ concentrations.


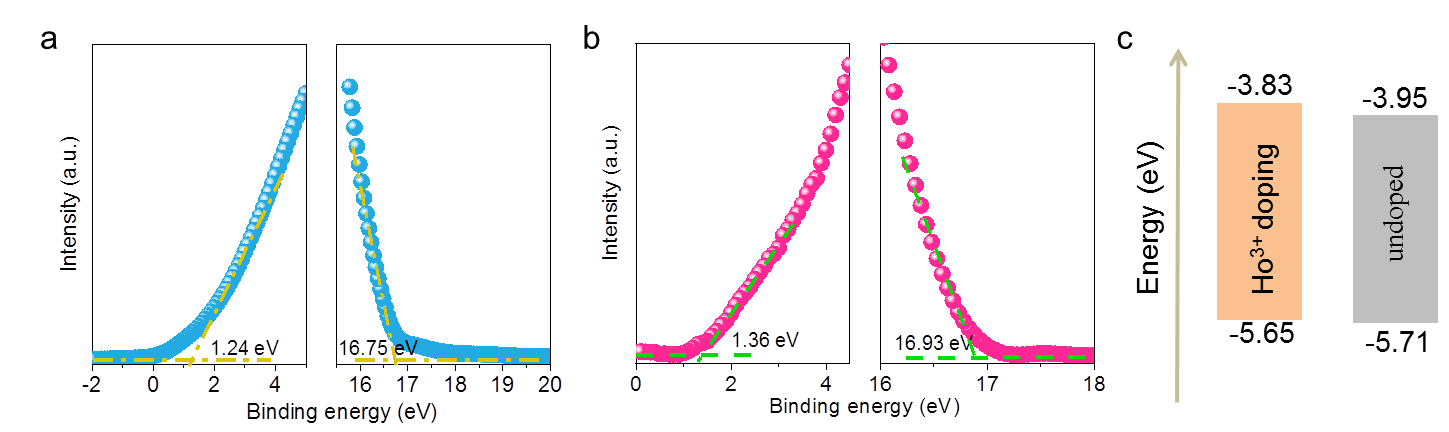


**Figure S11.** (a-b) The UPS curves of undoped CsPbI_3_ and CsPbI_3_: Ho^3+^ PQDs. (c) Energy band schematic of undoped CsPbI_3_ and CsPbI_3_: Ho^3+^ PQDs.


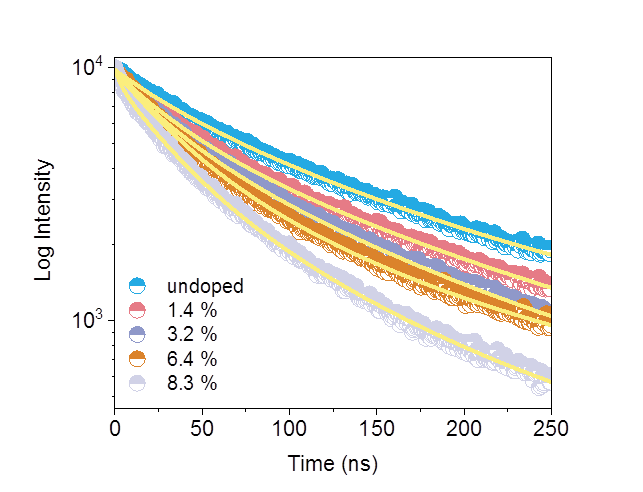


**Figure S12.** PL lifetimes of undoped CsPbI_3_ and Ho^3+^ doped CsPbI_3_ PQDs with different doping concentrations (0 %, 1.4 %, 3.2 %, 6.4 % and 8.3 %).


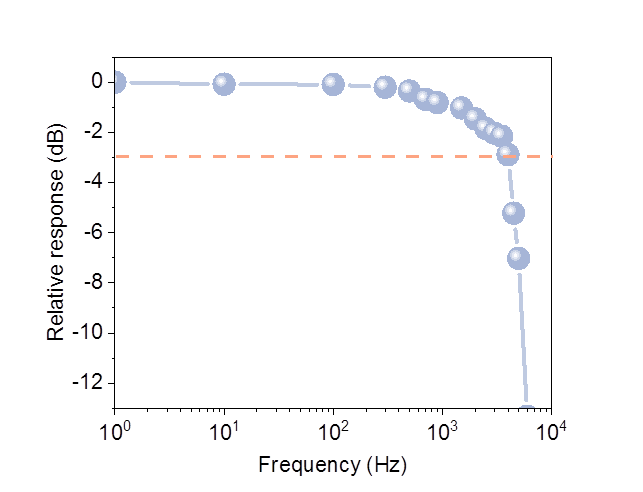


**Figure S13.** Temperature-dependent PL intensity of CsPbI_3_:Ho^3+^ (6.4 %) PQDs.


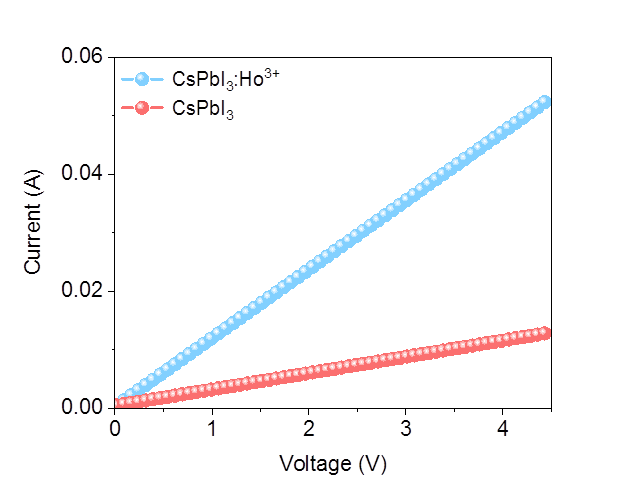


**Figure S14.** *I-V* curves of CsPbI_3_ and CsPbI_3_:Ho^3+^ (6.4 %) PQDs in FTO / PQDs /Ag devices.


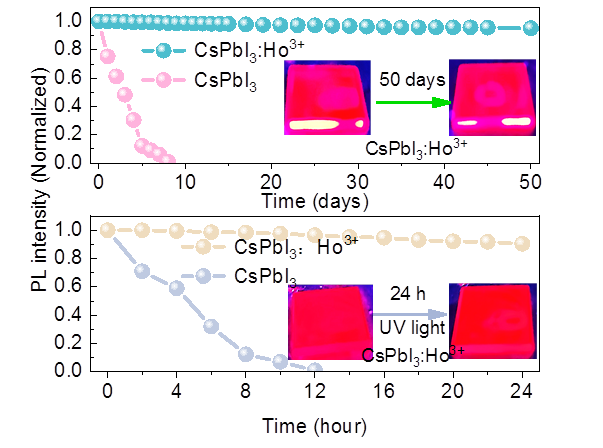


**Figure S15.** Storage time and UV light stability of CsPbI_3_ and CsPbI_3_:Ho^3+^ (6.4 %) PQDs; Inset is PL images of CsPbI_3_:Ho^3+^ PQDs film under air and UV light after 50 days and 24 h, respectively.


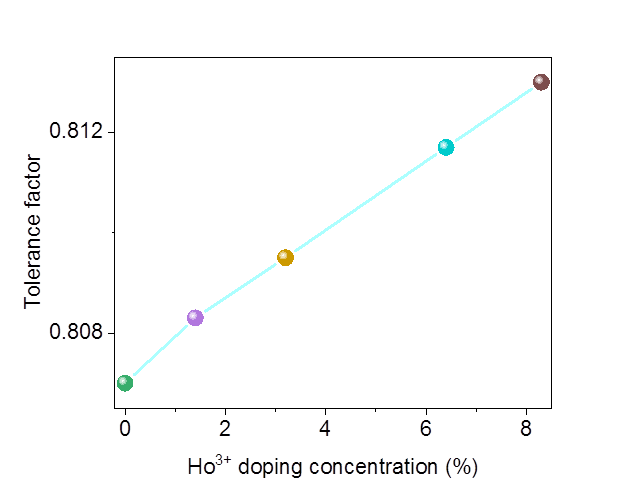


**Figure S16.** Effective Goldschmidt tolerance factors of undoped CsPbI_3_ and Ho^3+^ doped CsPbI_3_ PQDs with different doping concentrations (0 %, 1.4 %, 3.2 %, 6.4 % and 8.3 %).


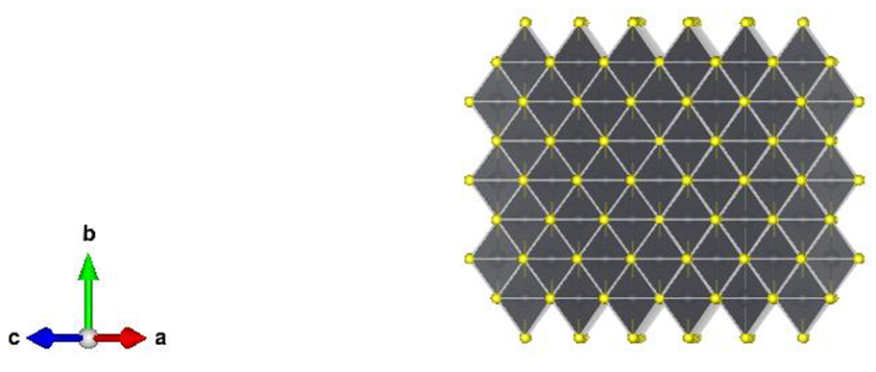


**Figure S17.** Structure diagram of PbS QDs.


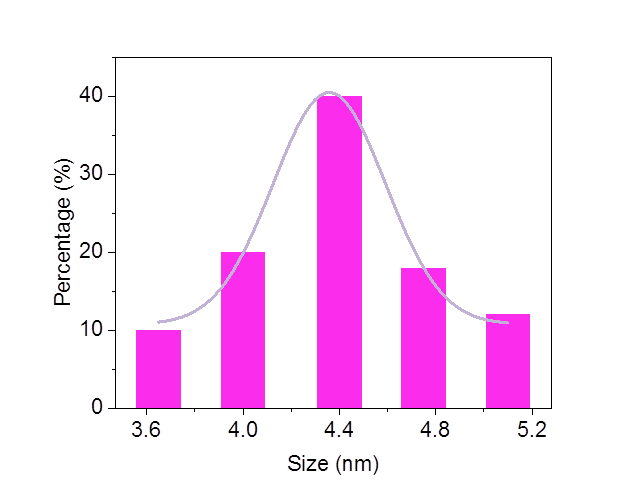


**Figure S18.** Size of PbS QDs.


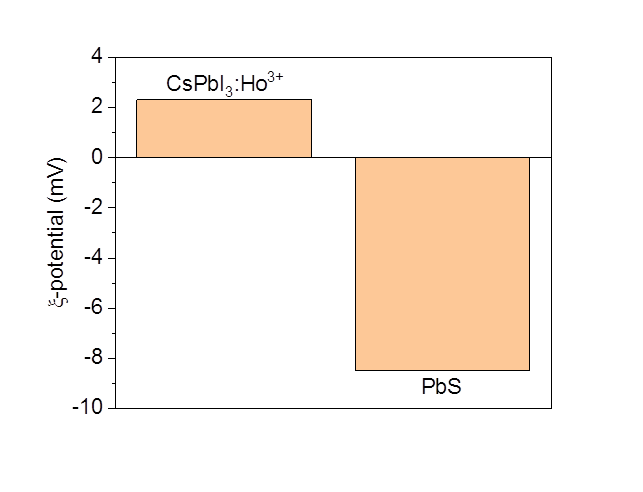


**Figure S19.** Variations in the zeta (*ζ*) potentials of the PbS QDs and CsPbI_3_:Ho^3+^ (6.4 %) PQDs.


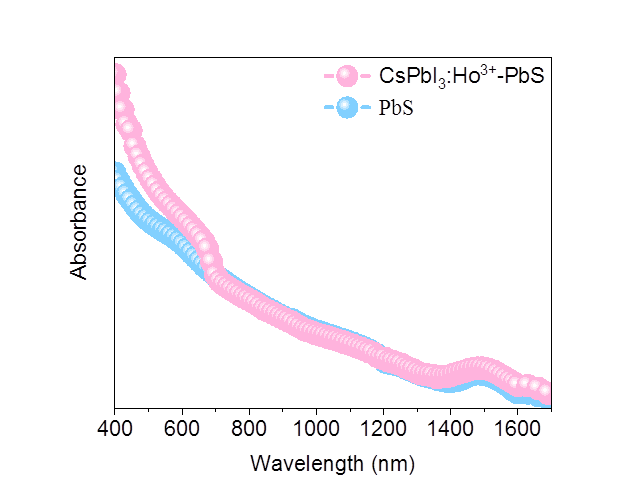


**Figure S20.** Absorption spectra of PbS QDs and CsPbI_3_:Ho^3+^- PbS QDs.


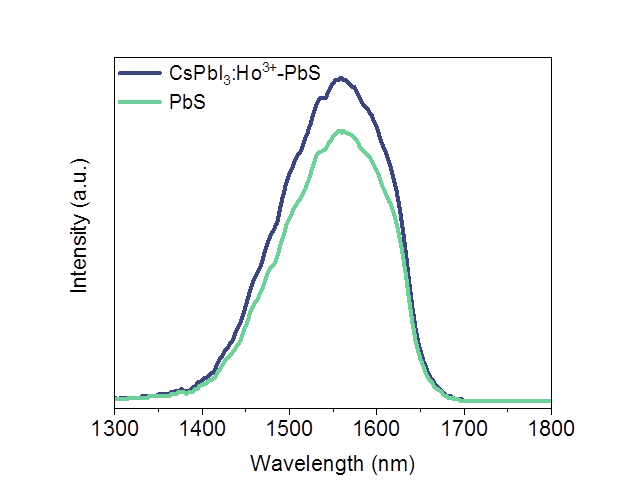


**Figure S21.** NIR emission of PbS QDs and CsPbI_3_:Ho^3+^-PbS QDs under 808 nm light.


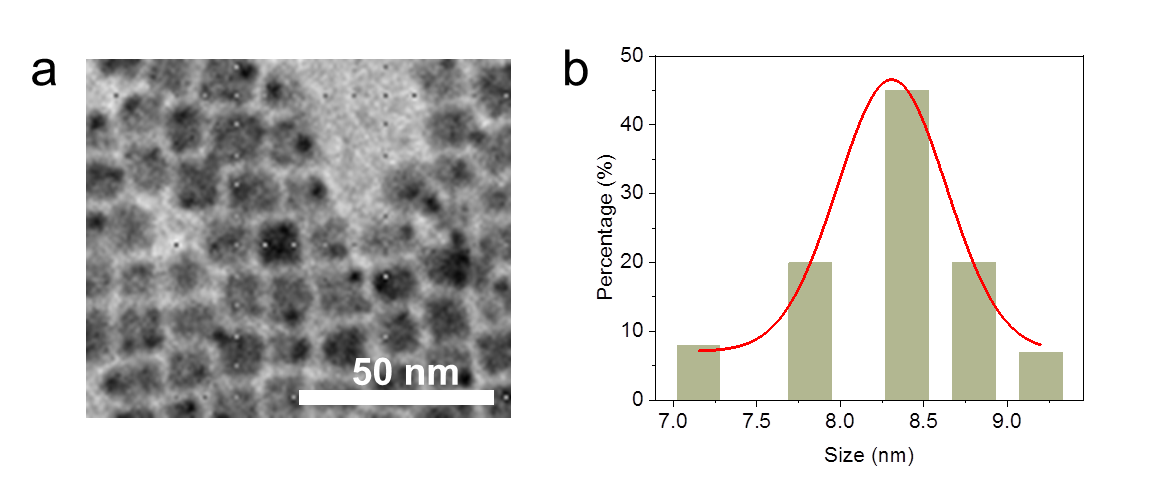


**Figure S22.** TEM and size images of the Cr^3+^/Ce^3+^/Yb^3+^/Er^3+^ doped CsPbCl_3_ PQDs.


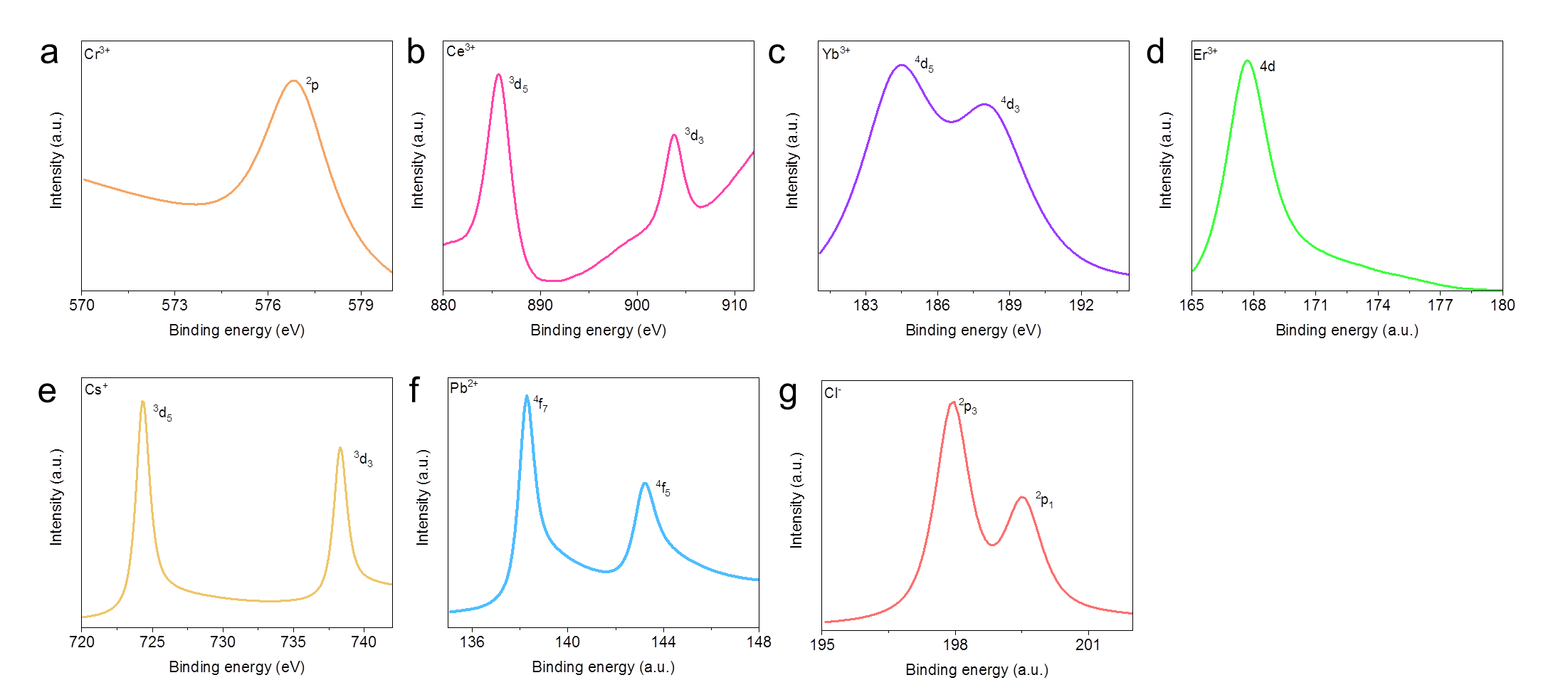


**Figure S23.** (a-g) XPS spectra of Cr (2 *p*), Ce (3 *d*), Yb(4 *d*), Er(4 *d*), Cs(3 *d*), Pb(4 *f*) and Cl(2 *p*) of Cr^3+^ / Ce^3+^ / Yb^3+^ / Er^3+^ doped CsPbCl_3_ PQDs.


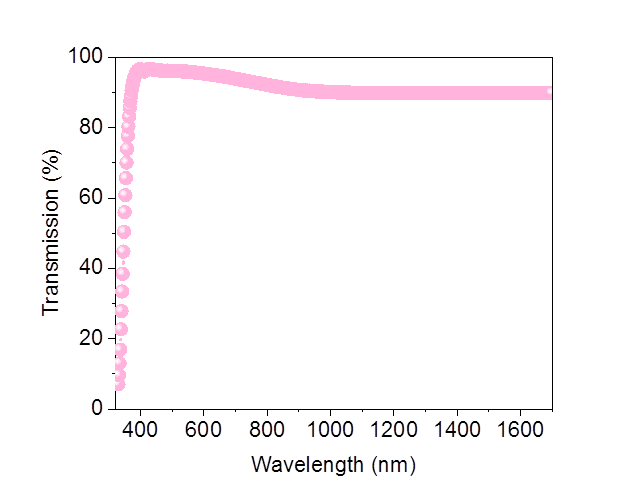


**Figure S24.** Transmission image of the QC-LC.


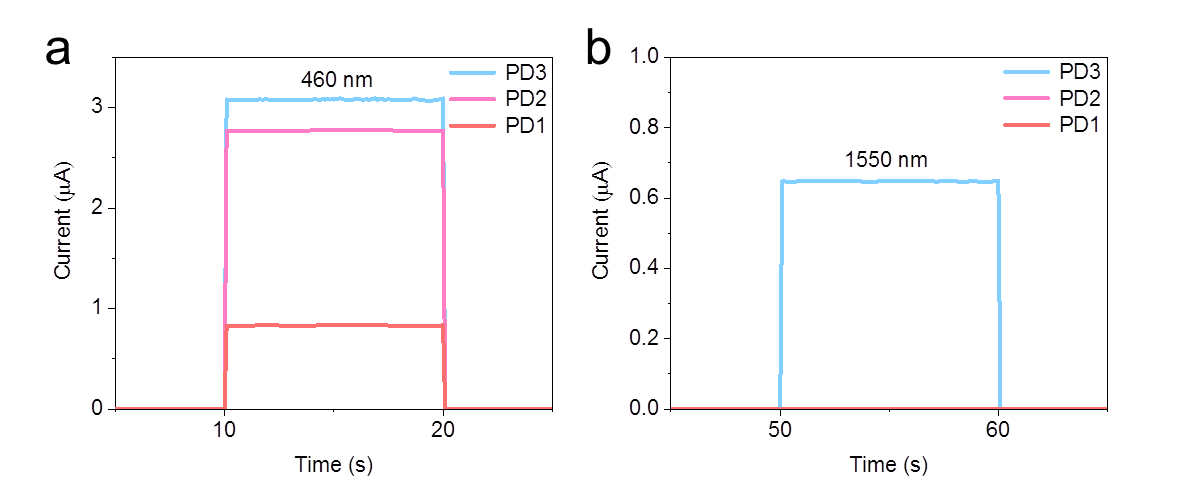


**Figure S25.** Photocurrents of PD1-PD3 under the 460 nm and 1550 nm light, respectively.


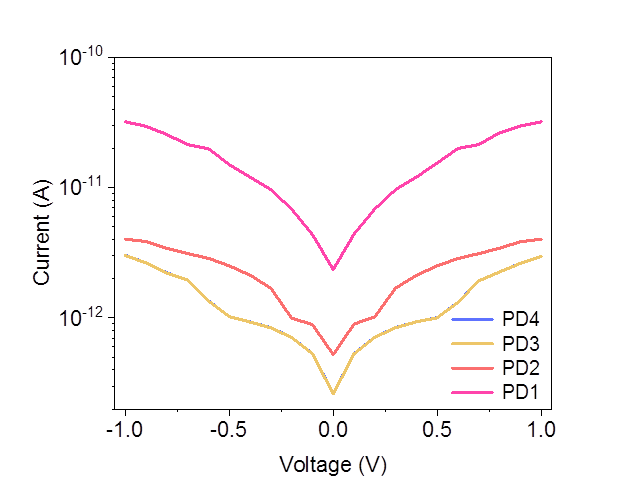


**Figure S26.** The I_d_ curves of PD1-PD4.


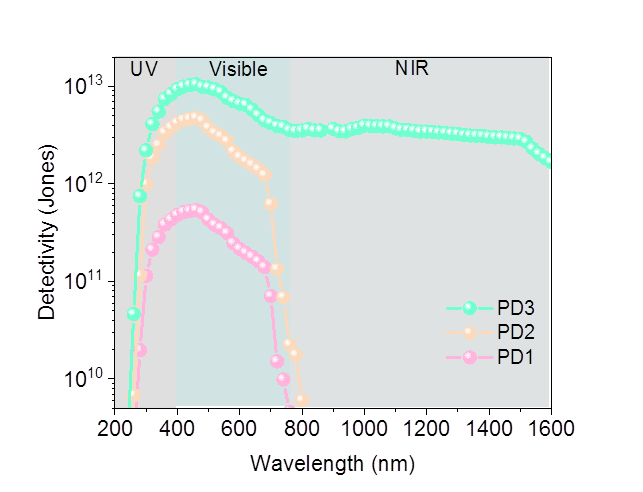
.

**Figure S27.** Detectivity of PD1-PD3.


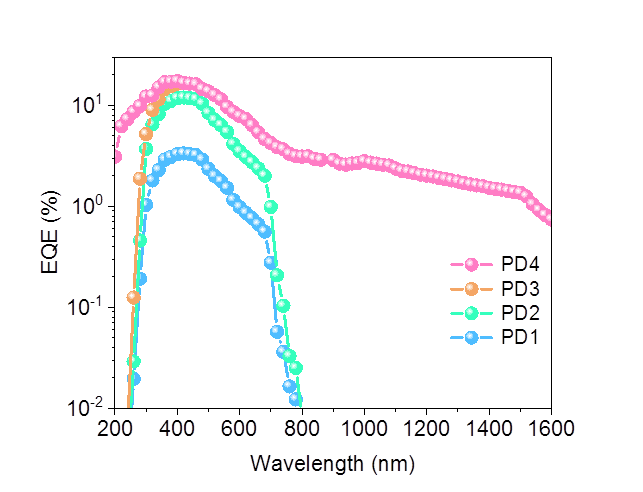


**Figure S28.** EQE of PD1-PD4.


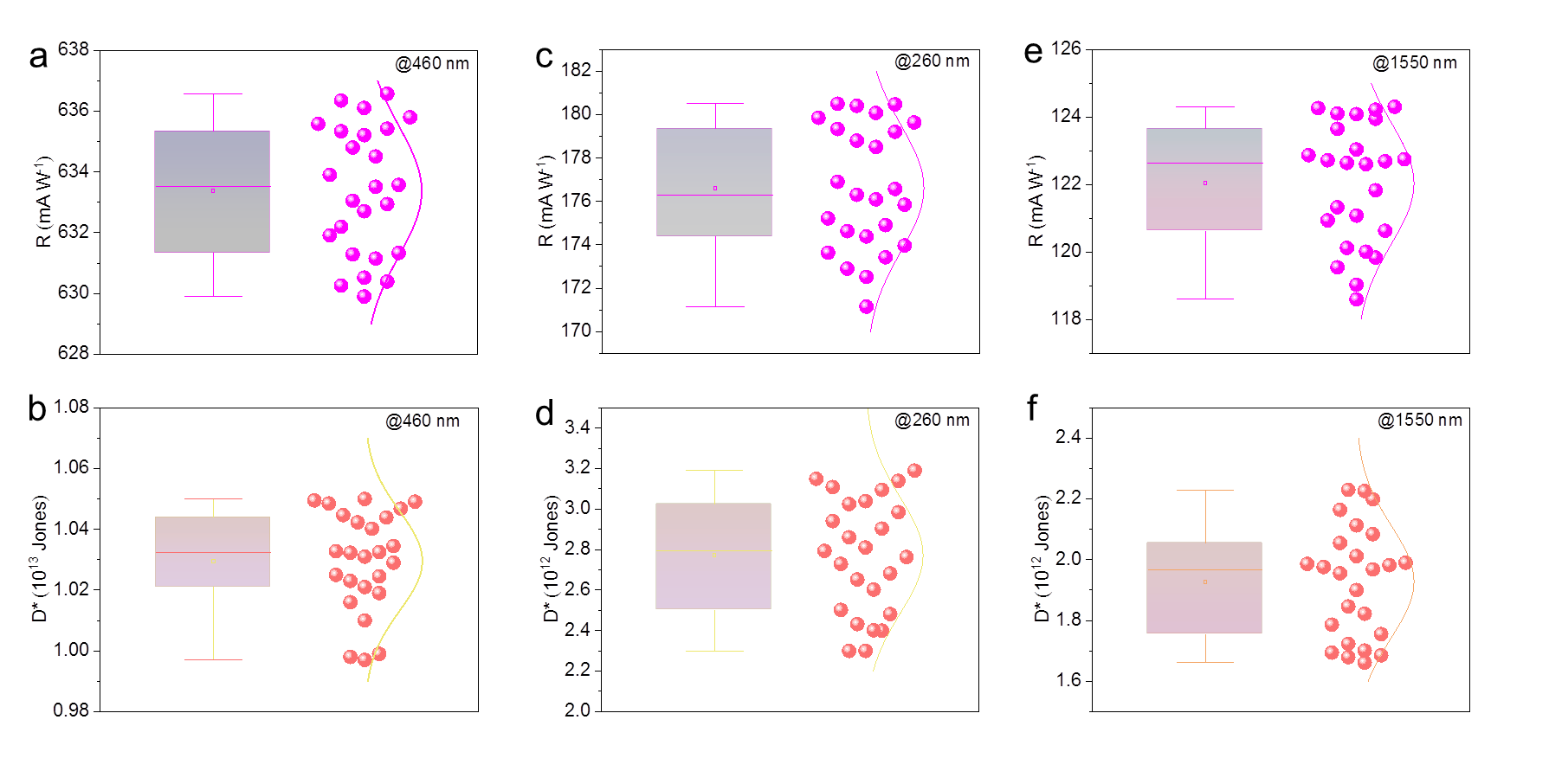


**Figure S29.** Averaged maximum R and D* of PD4 under 460 nm, 260 nm, and 1550 nm, respectively.


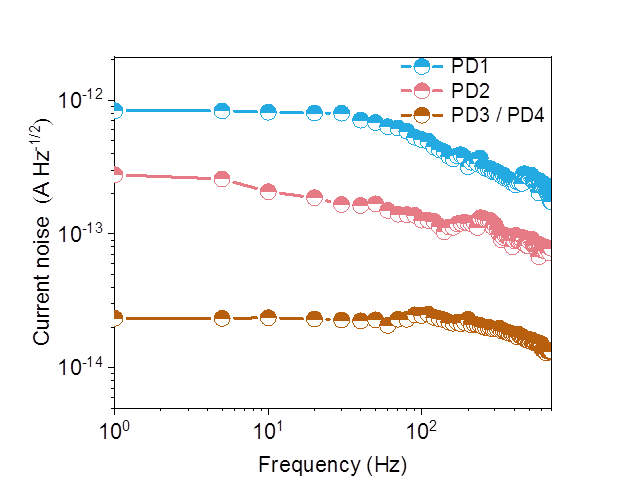


**Figure S30.** Noise current of the PD1-PD4.


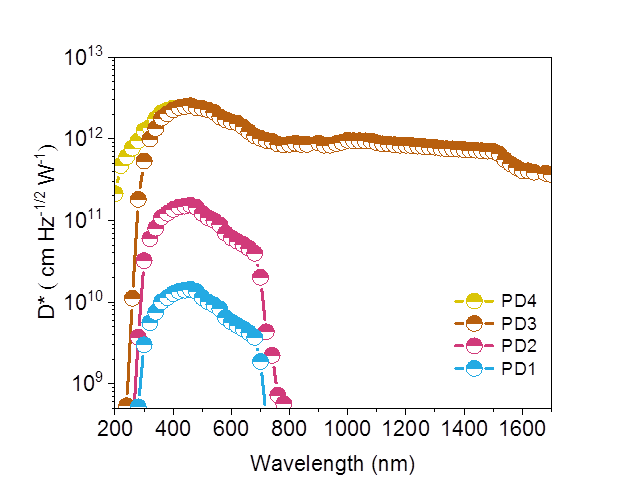


**Figure S31.** D* of PD1-PD4 based on the noise current.


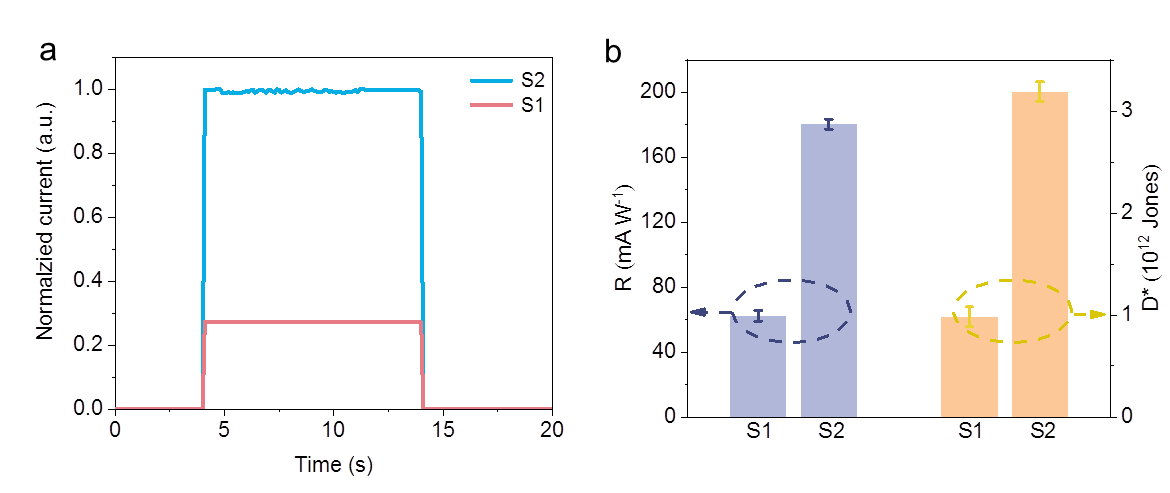


**Figure S32.** (a) Normalized photocurrents of PMMA: CsPbCl_3_:Cr^3+^,Yb^3+^, Er^3+^,Ce^3+^ PQDs / FTO / SnO_2_ / CsPbI_3_:Ho^3+^ - PbS QDs / MoO_3_ / Au (S1) and QC-LC / FTO / SnO_2_ / CsPbI_3_:Ho^3+^ - PbS QDs / MoO_3_ / Au (S2) under the 260 nm. (b) R and D* of S1 and S2 under the 260 nm, respectively.


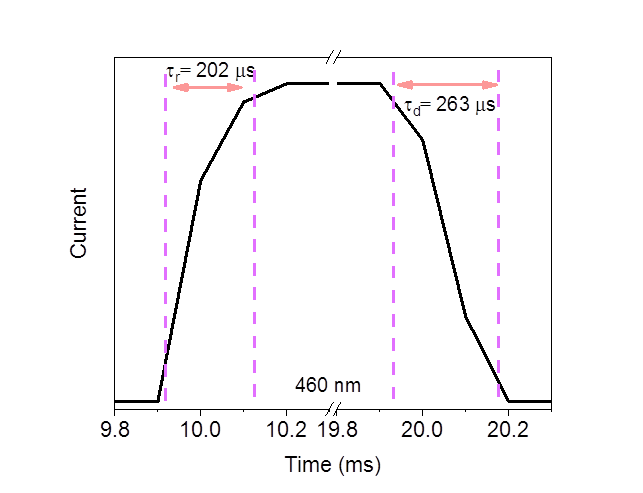


**Figure S33.** Evaluation of rising time and decay time of PD1 under 460 nm illumination.


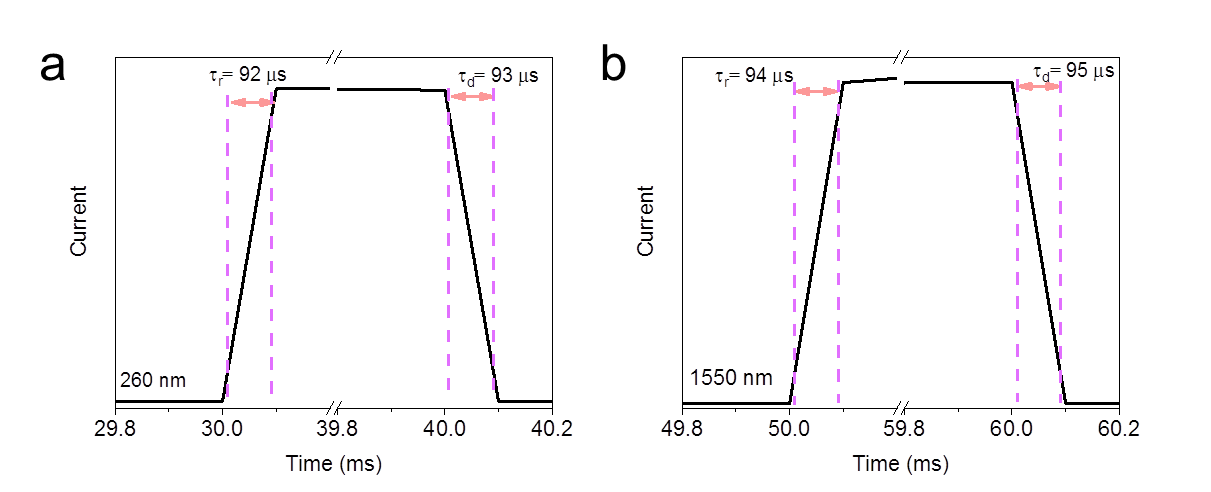


**Figure S34.** Evaluation of rising time and decay time of PD4 under 260 nm (a) and 1550 nm (b) illumination.


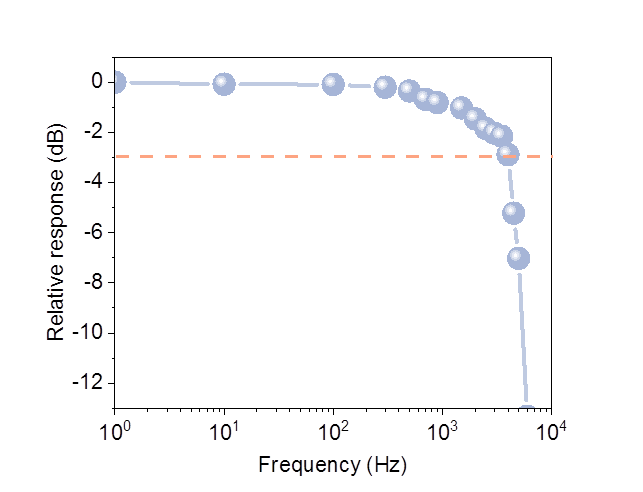


**Figure S35.** Relative response versus incident light modulation frequency for PD4.


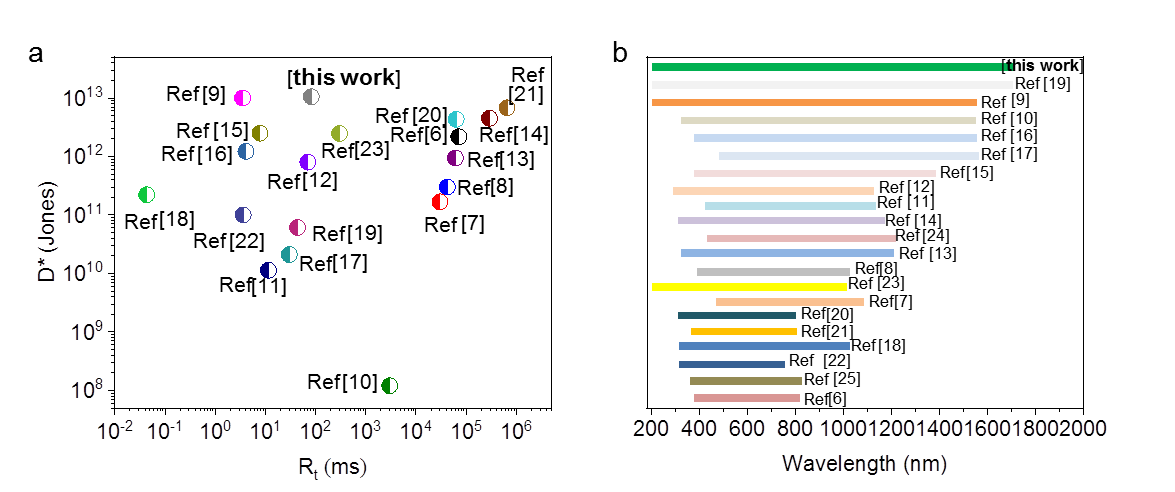


**Figure S36.** Comparison of D* and R_t_ (a), and response wavelength (b) among reported broadband PDs.


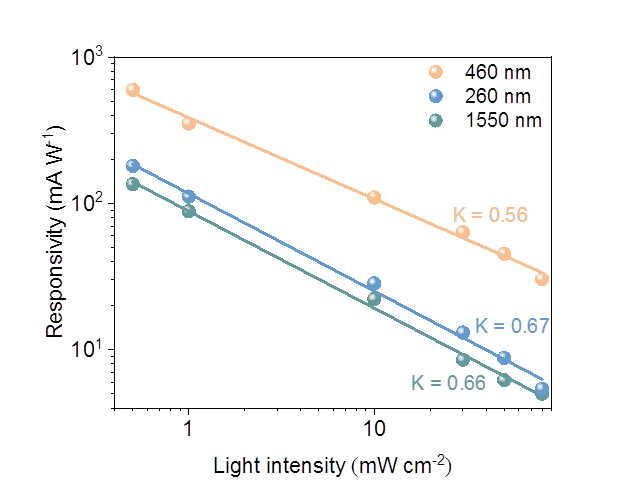


**Figure S37.** Responsivity versus light intensity for PD4 under 260 nm, 460 nm, and 1550 nm illumination, respectively.

**Table S1.** ICP-OES and XPS analysis of Ho^3+^ codoped CsPbI_3_ film with molar ratios of HoI_3_:PbI_2_ in the synthesis.


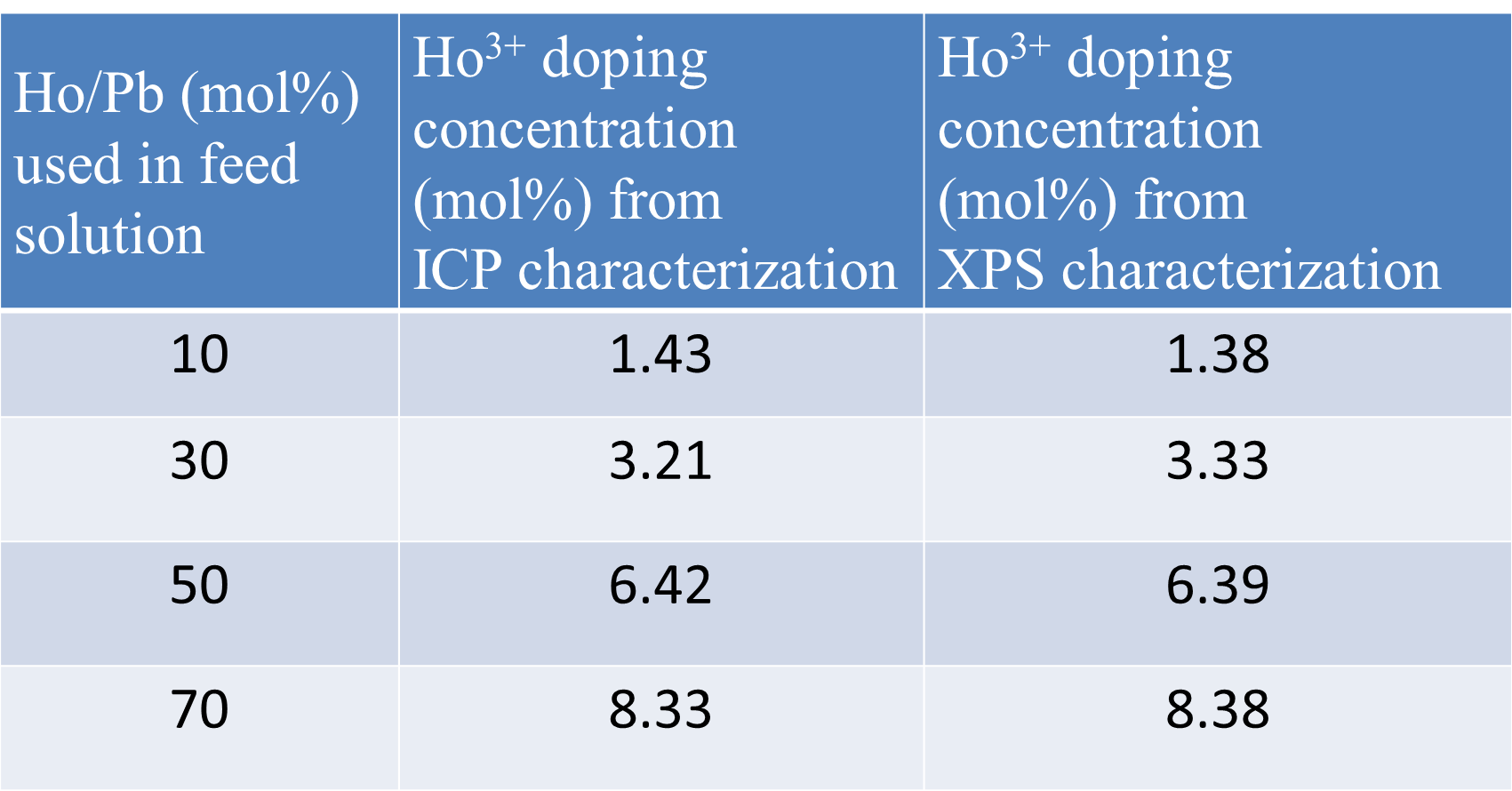


**Table S2.** Parameters of radiative decay rate, nonradiative decay rate, and ratios undoped and Ho^3+^ doped CsPbI_3_ PQDs as a function of Ho^3+^ doping concentration.


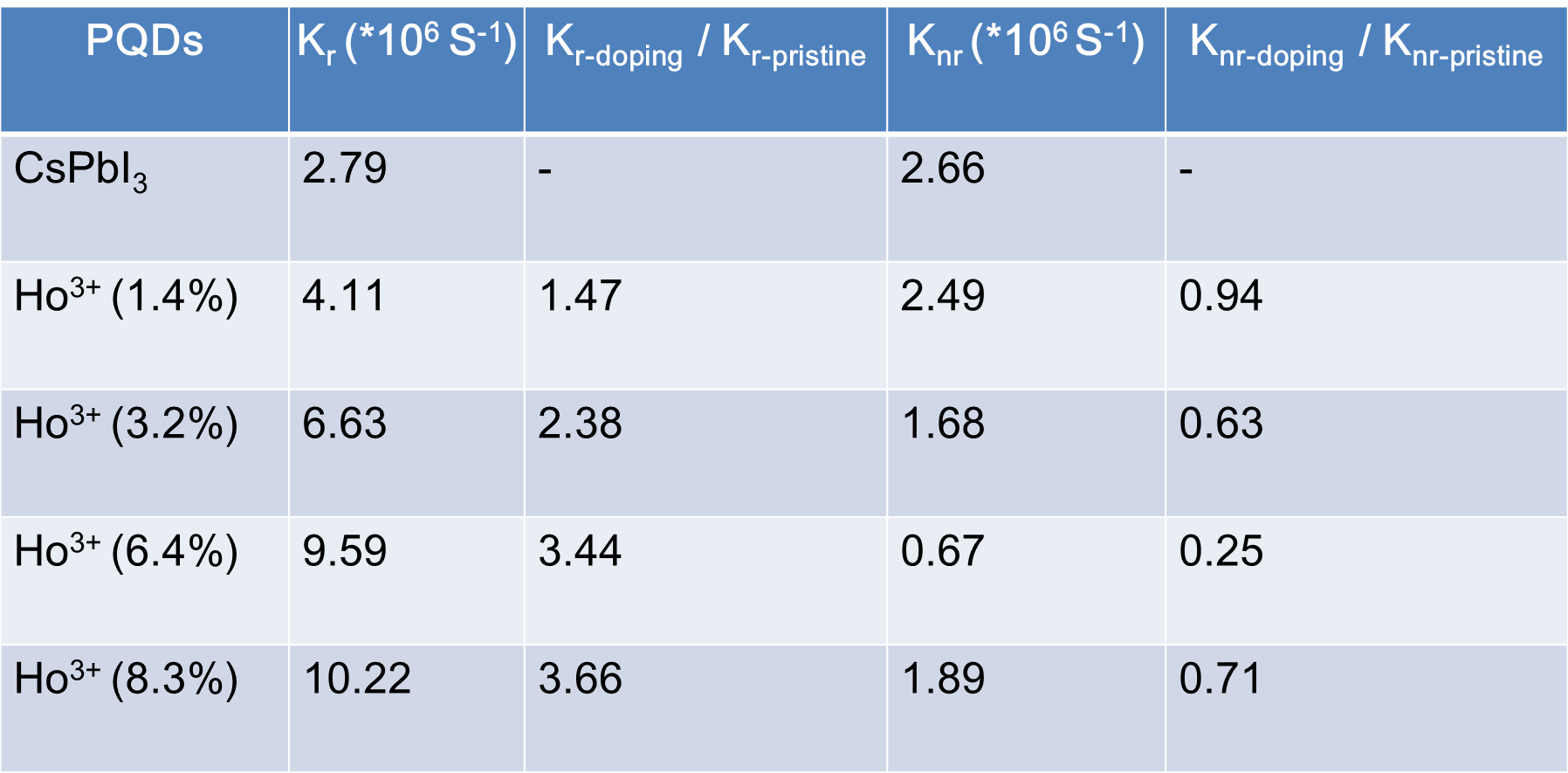


**References:**

[1] Brus, L. E. [A simple model for the ionization potential, electron affinity, and aqueous redox potentials of small semiconductor crystallites](https://pubs.aip.org/aip/jcp/article-abstract/79/11/5566/446897). *The Journal of chemical physics* **79**, 5566-5571 (1983).

### [2] Chen, C. et al. [Chemical inhibition of reversible decomposition for efficient and super-stable perovskite solar cells](https://www.sciencedirect.com/science/article/pii/S2211285519310225). *Nano Energy* **68**, 104315 (2020).

### [3] Fei, C. J. et al. [Self-assembled propylammonium cations at grain boundaries and the film surface to improve the efficiency and stability of perovskite solar cells](https://pubs.rsc.org/en/content/articlehtml/2019/ta/c9ta01755k). *Journal of Materials Chemistry A* **7**, 23739 (2019).

### [4] Shai, X. et al. [Achieving ordered and stable binary metal perovskite via strain engineering](https://www.sciencedirect.com/science/article/pii/S2211285518301873). *Nano Energy* **48**, 117 (2018).

### [5] Yu, W. et al. [Near‐Infrared Photodetectors Based on MoTe](https://onlinelibrary.wiley.com/doi/abs/10.1002/smll.201700268)_[2](https://onlinelibrary.wiley.com/doi/abs/10.1002/smll.201700268)_[/Graphene Heterostructure with High Responsivity and Flexibility](https://onlinelibrary.wiley.com/doi/abs/10.1002/smll.201700268). *Small* **13**, 1700268 (2017).

[6] Xia, H. et al. [Flexible and air-stable perovskite network photodetectors based on CH3NH3PbI3/C8BTBT bulk heterojunction](https://www.x-mol.com/paperRedirect/700247)**.** *Applied Physics Letters* **112**, 233301 (2018).

### [7] Qu, J. et al. [Space‐Confined Growth of Ultrathin P-Type GeTe Nanosheets for Broadband Photodetectors](https://onlinelibrary.wiley.com/doi/abs/10.1002/smll.202309391). *Small*. 2309391 (2024).

### [8] Zhang, J. Y. et al. [Toward broadband imaging: surface-engineered PbS quantum dot/perovskite composite integrated ultrasensitive photodetectors](https://pubs.acs.org/doi/abs/10.1021/acsami.9b14645). *ACS Appl. Mater. Interfaces* **11**, 44430-44437 (2019).

### [9] Zeng, L. H. et al. [Multilayered PdSe](https://onlinelibrary.wiley.com/doi/abs/10.1002/advs.201901134)_[2](https://onlinelibrary.wiley.com/doi/abs/10.1002/advs.201901134)_[/Perovskite Schottky Junction for Fast, Self‐Powered, Polarization‐Sensitive, Broadband Photodetectors, and Image Sensor Application](https://onlinelibrary.wiley.com/doi/abs/10.1002/advs.201901134). *Adv. Sci.* **6**, 1901134 (2019).

### [10] Yu, Y. et al. [Broadband Phototransistor Based on CH](https://pubs.acs.org/doi/abs/10.1021/acs.jpclett.6b02423)_[3](https://pubs.acs.org/doi/abs/10.1021/acs.jpclett.6b02423)_[NH](https://pubs.acs.org/doi/abs/10.1021/acs.jpclett.6b02423)_[3](https://pubs.acs.org/doi/abs/10.1021/acs.jpclett.6b02423)_[PbI](https://pubs.acs.org/doi/abs/10.1021/acs.jpclett.6b02423)_[3](https://pubs.acs.org/doi/abs/10.1021/acs.jpclett.6b02423)_[Perovskite and PbSe Quantum Dot Heterojunction](https://pubs.acs.org/doi/abs/10.1021/acs.jpclett.6b02423). *The journal of physical chemistry letters* **8**, 445-451 (2017).

### [11] Zhao, H. et al. [Self-driven visible-near infrared photodetector with vertical CsPbBr3/PbS quantum dots heterojunction structure](https://iopscience.iop.org/article/10.1088/1361-6528/ab4b17/meta). *Nanotechnology* **31**, 35202 (2020).

### [12] Tsai, M. et al. [Omnidirectional harvesting of weak light using a graphene quantum dot-modified organic/silicon hybrid device](https://pubs.acs.org/doi/abs/10.1021/acsnano.6b08567). *ACS Nano* **11**, 4564-4570 ( 2017).

### [13] Wang, M. et al. [Moisture‐triggered self‐healing flexible perovskite photodetectors with excellent mechanical stability](https://onlinelibrary.wiley.com/doi/abs/10.1002/adma.202100625). *Advanced Materials* **33**, 2100625 (2021).

### [14] Zhang, Z. et al. [High-performance broadband flexible photodetector based on Gd](https://www.nature.com/articles/s41378-023-00548-6)_[3](https://www.nature.com/articles/s41378-023-00548-6)_[Fe](https://www.nature.com/articles/s41378-023-00548-6)_[5](https://www.nature.com/articles/s41378-023-00548-6)_[O](https://www.nature.com/articles/s41378-023-00548-6)_[12](https://www.nature.com/articles/s41378-023-00548-6)_[-assisted double van der Waals heterojunctions](https://www.nature.com/articles/s41378-023-00548-6). *Microsystems & Nanoengineering* **9**, 84 (2023).

### [15] Liu, P. et al. [Double‐ended passivator enables dark‐current‐suppressed colloidal quantum dot photodiodes for CMOS‐integrated infrared imagers](https://onlinelibrary.wiley.com/doi/abs/10.1002/inf2.12497). *InfoMat* **6**, e12497 (2023).

### [16] Dang L Y. et al. [Efficient Carrier Transport in 2D Bi](https://onlinelibrary.wiley.com/doi/abs/10.1002/smll.202306600)_[2](https://onlinelibrary.wiley.com/doi/abs/10.1002/smll.202306600)_[O](https://onlinelibrary.wiley.com/doi/abs/10.1002/smll.202306600)_[2](https://onlinelibrary.wiley.com/doi/abs/10.1002/smll.202306600)_[Se/CsBi](https://onlinelibrary.wiley.com/doi/abs/10.1002/smll.202306600)_[3](https://onlinelibrary.wiley.com/doi/abs/10.1002/smll.202306600)_[I](https://onlinelibrary.wiley.com/doi/abs/10.1002/smll.202306600)_[10](https://onlinelibrary.wiley.com/doi/abs/10.1002/smll.202306600)_[Perovskite Heterojunction Enables Highly‐Sensitive Broadband Photodetection](https://onlinelibrary.wiley.com/doi/abs/10.1002/smll.202306600).*Small* **20**, 2306600 (2023).

### [17] Lee, C. H. et al. [Design of p-WSe](https://onlinelibrary.wiley.com/doi/abs/10.1002/adfm.202107992)_[2](https://onlinelibrary.wiley.com/doi/abs/10.1002/adfm.202107992)_[/n-Ge Heterojunctions for High-Speed Broadband Photodetectors](https://onlinelibrary.wiley.com/doi/abs/10.1002/adfm.202107992).

*Advanced Functional Materials* **32**, 2107992 (2021).

### [18] Ma, N. et al. [Stable and sensitive tin-lead perovskite photodetectors enabled by azobenzene derivative for near-infrared acousto-optic conversion communications](https://www.sciencedirect.com/science/article/pii/S2211285521003694). *Nano Energy* **86**, 106113 (2021).

### [19] Wang, Y. et al. [A room-temperature near-infrared photodetector based on a MoS 2/CdTe p–n heterojunction with a broadband response up to 1700 nm](https://pubs.rsc.org/en/content/articlehtml/2018/tc/c8tc01237g). *Journal of Materials Chemistry C* **6**, 4861-4865 (2018).

### [20] Wu, J. et al. [Constructing High‐Performance Solar Cells and Photodetectors with a Free Polythiophene Hole Transport Material](https://onlinelibrary.wiley.com/doi/abs/10.1002/adfm.202308584). *Advanced Functional Materials* **34**, 2308584 (2024).

### [21] Zhao, X. et al. [Enhanced photodetection of perovskite nanoplatelet devices by vertically stacked PEDOT: PSS/PbS/CsPbCl3 architecture](https://www.sciencedirect.com/science/article/pii/S0167577X21001634). *Materials Letters* 290, 129467 (2021).

### [22] He, J. et al. [Improving photoelectric conversion with broadband perovskite metasurface](https://pubs.acs.org/doi/abs/10.1021/acs.nanolett.2c01979). *Nano Letters* **22**, 6655-6663 (2022).

### [23] Ding, N. et al. [A novel approach for designing efficient broadband photodetectors expanding from deep ultraviolet to near infrared](https://www.nature.com/articles/s41377-022-00777-w). *Light: Sci. Appl.* **11**, 91 (2022).

### [24] Zhao, X. et al. [Vertically stacked PEDOT: PSS/PbS/CsPbCl](https://www.sciencedirect.com/science/article/pii/S0925838821004047)_[3](https://www.sciencedirect.com/science/article/pii/S0925838821004047)_ [for flexible optoelectronic devices](https://www.sciencedirect.com/science/article/pii/S0925838821004047). *Journal of Alloys and Compound*s **866**, 158997 (2021).

### [25] Yao, J. et al. [All‐Layered 2D Optoelectronics: A High‐Performance UV–vis–NIR Broadband SnSe Photodetector with Bi](https://onlinelibrary.wiley.com/doi/abs/10.1002/adfm.201701823)_[2](https://onlinelibrary.wiley.com/doi/abs/10.1002/adfm.201701823)_[Te](https://onlinelibrary.wiley.com/doi/abs/10.1002/adfm.201701823)_[3](https://onlinelibrary.wiley.com/doi/abs/10.1002/adfm.201701823)_[Topological Insulator Electrodes](https://onlinelibrary.wiley.com/doi/abs/10.1002/adfm.201701823). *Advanced Functional Materials* **27**, 1701823 (2017).
